# The thirsty fly: Ion transport peptide (ITP) is a novel endocrine regulator of water homeostasis in *Drosophila*

**DOI:** 10.1371/journal.pgen.1007618

**Published:** 2018-08-23

**Authors:** Martina Gáliková, Heinrich Dircksen, Dick R. Nässel

**Affiliations:** Department of Zoology, Stockholm University, Stockholm, Sweden; Washington University in Saint Louis School of Medicine, UNITED STATES

## Abstract

Animals need to continuously adjust their water metabolism to the internal and external conditions. Homeostasis of body fluids thus requires tight regulation of water intake and excretion, and a balance between ingestion of water and solid food. Here, we investigated how these processes are coordinated in *Drosophila melanogaster*. We identified the first thirst-promoting and anti-diuretic hormone of *Drosophila*, encoded by the gene *Ion transport peptide (ITP)*. This endocrine regulator belongs to the CHH (crustacean hyperglycemic hormone) family of peptide hormones. Using genetic gain- and loss-of-function experiments, we show that *ITP* signaling acts analogous to the human vasopressin and renin-angiotensin systems; expression of *ITP* is elevated by dehydration of the fly, and the peptide increases thirst while repressing excretion, promoting thus conservation of water resources. *ITP* responds to both osmotic and desiccation stress, and dysregulation of *ITP* signaling compromises the fly’s ability to cope with these stressors. In addition to the regulation of thirst and excretion, *ITP* also suppresses food intake. Altogether, our work identifies ITP as an important endocrine regulator of thirst and excretion, which integrates water homeostasis with feeding of *Drosophila*.

## Author summary

Maintenance of energy and water balance is necessary for survival of all organisms. Even a mild dehydration triggers thirst, reduces appetite, and decreases diuresis (water excretion), thereby promoting conservation of water resources and survival under arid conditions. Homeostasis is regulated primarily by endocrine systems that utilize neuropeptides and peptide hormones. Whereas hormonal mechanisms that regulate the water balance in humans are relatively well understood, much less is known about these regulations in the fruit fly *Drosophila melanogaster*. Here, we describe the first thirst-promoting and anti-diuretic hormone of *Drosophila*, encoded by the gene *Ion transport peptide (ITP)*. We show that *ITP* increases upon dehydration, and protects the animal from loss of body water by promoting thirst and repressing excretion. *ITP* also suppresses feeding, and can thus be considered as a master regulator integrating water and energy balance.

## Introduction

Maintenance of homeostasis is based on ingestion and metabolism of water and nutrients in a manner that reflects the internal needs of the animal, but the precise regulatory mechanisms are incompletely understood [1]. Despite the strong evolutionary conservation of the main pathways underlying energy homeostasis [2–5], there is a considerable diversity in the strategies involved in the maintenance of water balance [6, 7]. In insects, this variability arises mainly from the diversity of their habitats and life history strategies. For example, some blood-sucking insects are able to ingest a blood meal that exceeds their body volume up to twelve-fold; their feeding is hence coupled to massive post-prandial diuresis of the excessive water and ions [8]. However, in most of the non-blood sucking terrestrial insects, water conservation is more important than water secretion [1, 9]. Studies on water balance in insects have historically focused mainly on the hormonal regulation of water excretion. These studies investigated the correlations between the hormone titers and diuresis, and analyzed the effects of injections or in vitro applications of the tested compounds (reviewed e.g. in [8–11]). These works contributed to a better understanding of water regulation at the level of fluid secretion by the Malpighian tubules and water reabsorption in the hindgut (reviewed e.g. in [8–11]). Later, development of genetic tools for *Drosophila* allowed analysis of diuretic hormones by direct genetic manipulations [12–14]. However, no anti-diuretic hormone has been identified in *Drosophila* until now.

*Drosophila* is under laboratory conditions raised on media that provide both nutrients and water, and flies therefore do not regulate food and water intake independently. Nevertheless, insects, including *Drosophila*, can sense water [15, 16] and exhibit hygrotactic behavior [17, 18]. If given the opportunity, flies differentiate between food and water sources, and are able to seek and drink free water [19, 20], or ingest media rich in water but devoid of nutrients [21]. Recently, a small group of neurons were identified in the *Drosophila* brain that antagonistically regulate thirst and hunger [22]. These neurons sense osmolarity cell-autonomously with the cation channel Nanchung, and internal nutrients indirectly via Adipokinetic hormone signaling [22]. Although several hormones have been shown to regulate feeding and satiety (reviewed in [23–27]), no endocrine regulator of thirst has been identified in *Drosophila* so far.

The mechanisms that orchestrate water sensing, water-seeking behavior and conservation of water remain unclear. We hypothesized that these processes are likely coordinated by endocrine signaling. Physiological roles of *Drosophila* hormones are mostly well characterized (reviewed e.g. in [23]); one of the few exceptions is Ion transport peptide (ITP), which belongs to the family of crustacean hyperglycemic hormones (CHH) [28, 29]. CHHs promote water reuptake and hence, act as an anti-diuretic hormones in crustaceans [30]. The locust homolog of ITP promotes water reabsorption by acting on chloride channels in the hindgut [31, 32]. *Drosophila* has a single *ITP* gene that gives rise to an amidated ITP hormone and to two longer forms called ITP-like peptides [28, 29]. The functions of *Drosophila ITP* have not been investigated so far, except for a study that has shown a role of *ITP* in modulation of evening activity by the circadian clock circuitry [33]. The findings from the crustacean [34] and locust [31, 32] members of the CHH family suggest that *Drosophila ITP* might be involved in the regulation of water balance as well. Here, we tested this hypothesis by investigating the effects of gain- and loss-of-function of *ITP* on key aspects of water homeostasis, such as body water content, desiccation and osmotic stress resistance, food and water intake, and excretion. Our work identified master regulatory roles of *ITP* in water homeostasis of *Drosophila*; *ITP* levels increase under desiccation stress and protect the fly from water loss by increasing thirst, reducing excretion rate, and promoting ingestion of water instead of food. Altogether, our work identifies the first anti-diuretic and drinking-promoting hormone in *Drosophila*, which also coordinates water balance with feeding behavior.

## Results

### *ITP* codes for an anti-diuretic peptide

**Fig 1:**
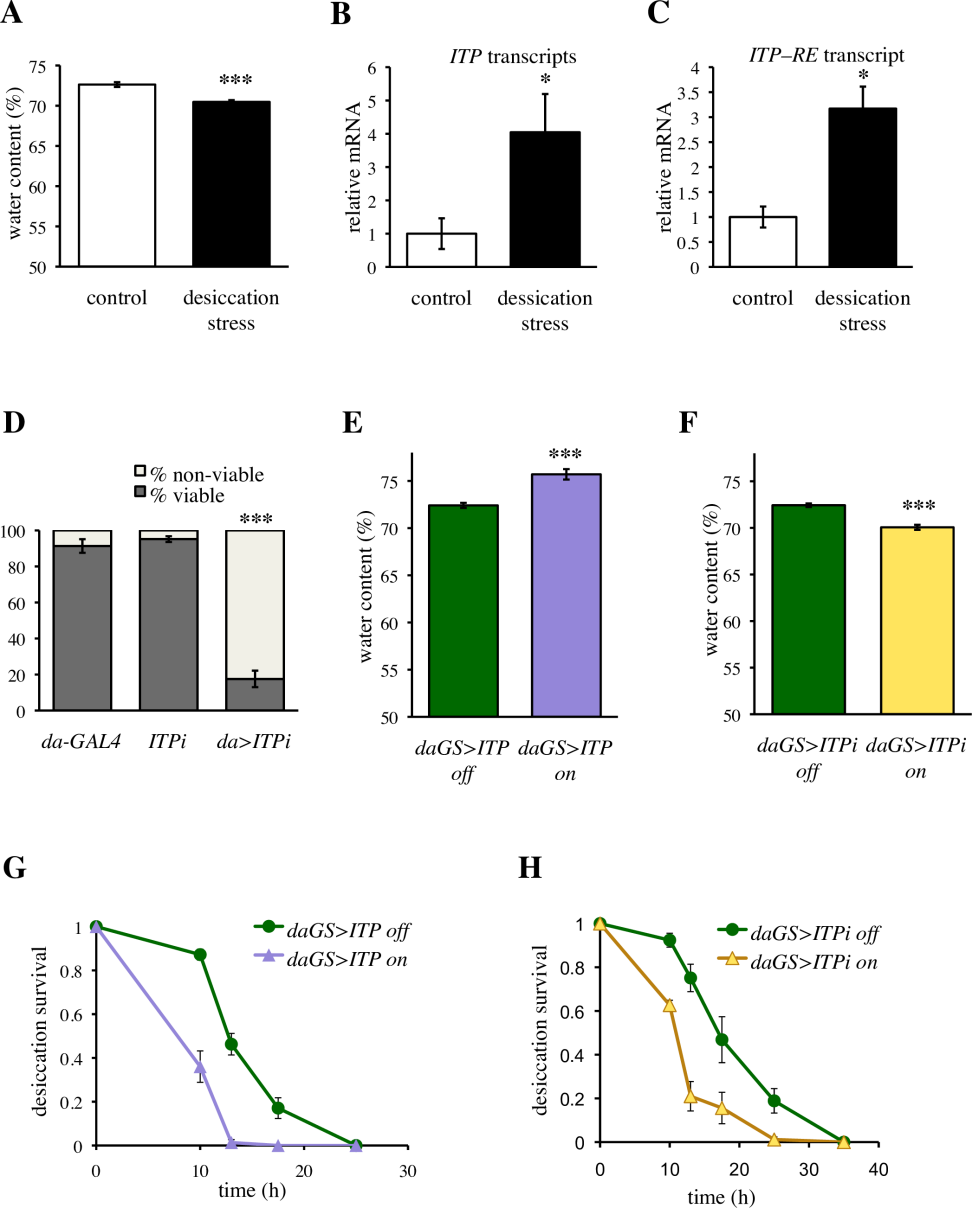
*ITP* regulates water homeostasis. (A) Short term (6 h) desiccation stress efficiently reduces body fluids. Two-tailed Student’s *t*–test: *P* < 0.001. (B) Desiccation stress increases expression of the *ITP* gene. A primer pair that detects all 5 isoforms (transcripts *RC*, *RD*, *RE*, *RF* and *RG*, FlyBase FB2017_06) was used. Two-tailed Student’s *t*–test: *P* < 0.05. (C) Desiccation stress increases the abundance of the *RE* transcript. Primer pair that detects solely this isoforms was used. Two-tailed Student’s *t*–test: *P* < 0.05. (D) *Daughterless*-GAL4 driven *ITP* RNAi *(da>ITPi)* results in developmental lethality. Fischer’s exact test: *P* < 0.001 for comparison with each control. Animals were analyzed in three replicates, Fischer’s exact test was done on pooled data. Sample size: *da-GAL4* n = 595; *ITPi* n = 439*; da>ITPi* n = 566. (E) *Daughterless-GeneSwitch*-driven over-expression of *ITP (daGS>ITP)* increases the proportion of body fluids. Two-tailed Student’s *t*–test: *P* < 0.001. (F) *daGS* driven *ITPi (daGS>ITPi)* decreases the proportion of body fluid. Two-tailed Student’s *t*–test: *P* < 0.001. (G) Over-expression of *ITP* decreases desiccation resistance. Log-rank test: *P* < 0.001. Sample size: *daGS>ITP* off n = 83; *daGS>ITP* on = 70. (H) *ITP* RNAi decreases desiccation resistance. Log-rank test: *P* < 0.001. Sample size: *daGS>ITPi* off n = 88; *daGS>ITPi* on n = 89.

As the first step towards understanding the potential role of *ITP* in water homeostasis of *Drosophila*, we investigated whether expression of this gene reflects changes in the body water. We exposed standard (*w*^*1118*^) flies to a short-term (6 h) desiccation, which was sufficient to reduce body fluids (Fig 1A), and monitored expression of the *ITP* gene (CG13586) by quantitative PCR. Using a primer pair that covers all 5 known transcripts of the gene, we showed that desiccation stress increases expression of the *ITP* gene (Fig 1B), suggesting a role of *ITP* in water homeostasis. We confirmed that the transcriptional increase involves also the *RE* transcript (Fig 1C), the only transcript that gives rise to ITP (FlyBase FB2017_06), considered to be the only functional peptide produced by the *ITP* gene [28].

It has been shown that an *ITP* mutation is embryonically lethal [35], and RNAi driven by the ubiquitous *daughterless*-GAL4 (*da*-GAL4) also resulted in considerable developmental lethality (Fig 1D). Therefore, to investigate the role of *ITP* in water balance, we used the GeneSwitch system [36, 37], which allowed circumventing the developmental lethality of *ITP* and studying the gain- and loss-of function of *ITP* specifically during the adult stage. In addition, this system enabled investigation of genetically identical animals, thereby avoiding any confounding effects of genetic backgrounds. The system is switched on by feeding flies the drug RU-486, which in itself does not affect water balance (S1 Fig). The expression pattern of *ITP* is complex and involves several distinct neuron types in the central nervous system and periphery, but the hormone is supposed to be released into the hemolymph [28, 29]. Therefore, we used the ubiquitous *daughterless*-GeneSwitch (*daGS*) [38] driver for both RNAi *(ITPi)* and over-expression of *ITP*. The *daGS*-driven over-expression of *ITP* resulted in increased (Fig 1E), and RNAi in decreased water content (Fig 1F), demonstrating that *ITP* has anti-diuretic function. We reproduced this effect also using an independent RNAi line targeting an alternative part of the *ITP* transcript (S2 Fig), and confirmed that *ITP* has anti-diuretic activity in female flies as well (S3 Fig). However, despite their higher initial water content, animals with increased *ITP* levels were more sensitive to desiccation (Figs 1G and S4), with their survival reduced by over 30%. Interestingly, animals with reduced *ITP* levels had moderately increased sensitivity to desiccation as well (Figs 1H and S4), suggesting that survival under arid conditions depends on a tightly regulated expression of *ITP*.

Taken together, these experiments revealed that *ITP* codes for a hormone that is regulated by internal water content and has an anti-diuretic function.

### *ITP* is required to cope with desiccation stress

**Fig 2:**
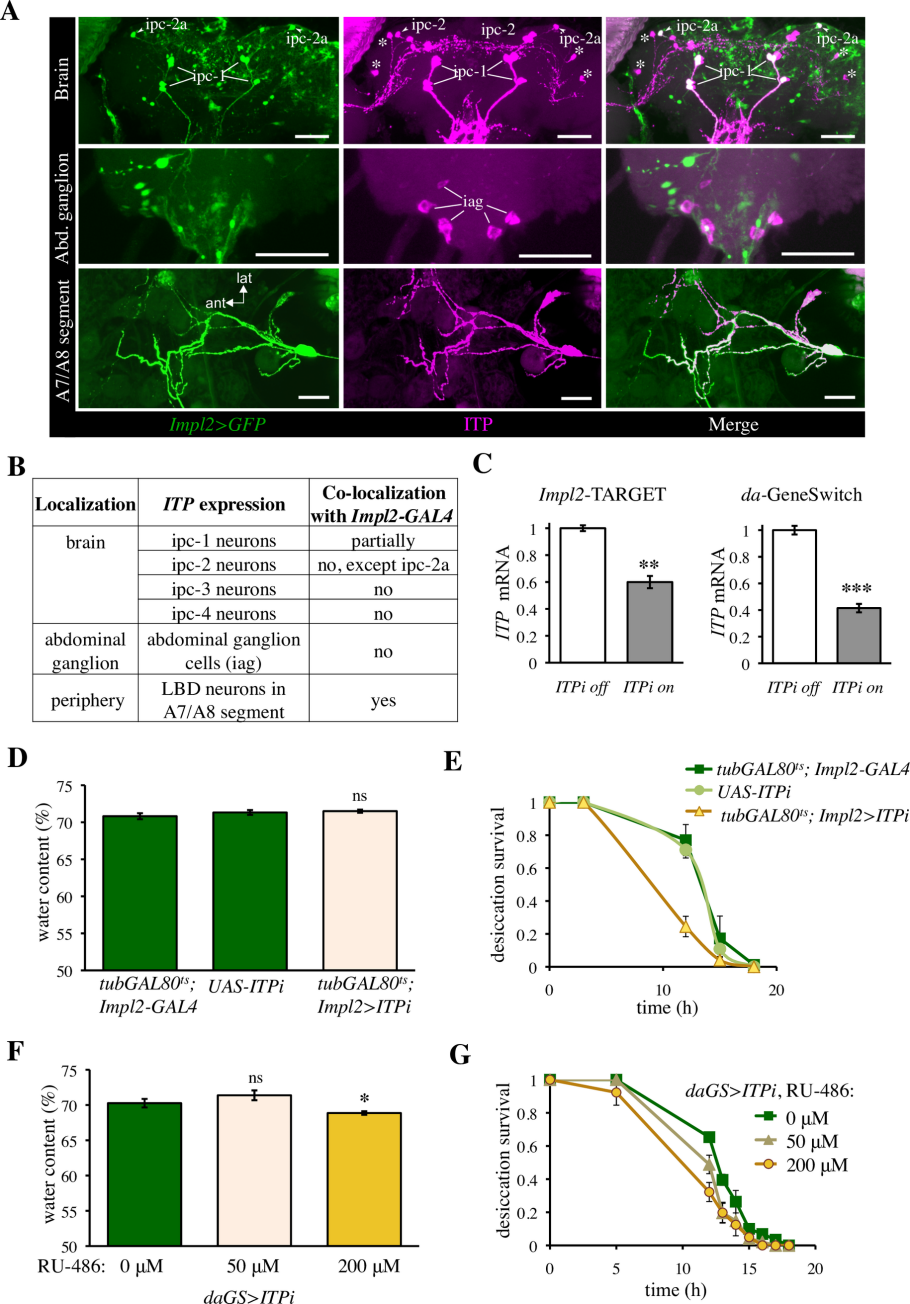
*ITP* regulates desiccation survival independently of its action on the water storage under ad libitum feeding. (A) *Impl2-GAL4* is a suitable driver to target several of the *ITP*-expressing neurons. *Impl2-GAL4*-driven expression of GFP partially overlaps with the ITP immunoreactivity (ITPir); scale bars 50 μm. Upper panel: brain neurons. Note the ipc-1 and ipc-2a neurosecretory cells are covered by the *Impl2* expression pattern, whereas the rest of the ipc-2 (marked in middle figure) and ipc-3 interneurons (asterisks) are not. Median panel: neurons in the terminal abdominal ganglia that express ITP do not express of *Impl2>GFP*. iag = ITPir abdominal ganglia neurons. Lower panel: peripheral lateral bipolar dendrite (LBD) neurons in the seventh and eighth abdominal segment express both *Impl2>GFP* and ITP (ant anterior, lat lateral). (B) Summary table listing partially overlapping expression pattern of *Impl2-GAL4* and ITP. (C) *ITPi* driven by both *Impl2*-based TARGET and *da-*GeneSwitch effectively decreases *ITP* mRNA. Two-tailed Student’s *t*–test: *P* < 0.01 for the TARGET manipulations and *P* < 0.001 for the GeneSwitch manipulation. F1 generation of the cross between the *ITPi* line and *w*^*1118*^ was used as a control (off conditions) for the TARGET-based experiment. (D) *ITPi* driven by the *Impl2*- based TARGET does not affect the proportion of body fluids. Two-tailed Student’s *t*–test: *P* > 0.05 for both comparisons with controls. (E) *ITPi* driven by the *Impl2*-based TARGET reduces survival under desiccation. Log-rank test: *P* < 0.001 for comparisons with both controls. Sample size: *tub-GAL80*^*ts*^*; Impl2-GAL4* n = 87; *ITPi* n = 83; *tub-GAL80*^*ts*^*; Impl2>ITPi* n = 49. (F) Mild *ITPi* driven by the *daGS* induced by 50 μM RU-486 does not affect body fluids. Two-tailed Student’s *t*–test: *P* > 0.05 for comparisons with the non-induced conditions. Strong *ITPi* driven by the *daGS* induced by 200 μM RU-486 reduces body water. Two-tailed Student’s *t*–test: *P* < 0.05. (G) Mild *ITPi* driven by the *daGS* induced by 50 μM RU-486 reduces survival under desiccation. Log-rank test: *P* < 0.05. The detrimental effect of *ITPi* on desiccation resistance is dose-dependent; induction by 50 μM RU-486 has a milder effect than induction by the 200 μM RU-486. Log-rank test: *P* < 0.01. Sample size: 0 μM RU-486 n = 61; 50 μM RU-486 n = 45; 200 μM RU-486 n = 40.

Next, we asked if *ITP* regulates the response to desiccation, or whether it determines desiccation resistance only by influencing the initial water content prior to the desiccation. The *daGS>ITPi* manipulations from the experiments described above could not answer these questions, because they resulted in reduced body water already before the onset of desiccation. Thus, we looked for a weaker genetic manipulation of ITP, which would allow testing the desiccation resistance without affecting the initial water content. Using an ITP-specific antibody, we confirmed previous results [28, 29] showing that the gene is expressed in the neurosecretory cells of the brain termed ipc-1 and ipc-2, in the interneurons termed ipc-3 and ipc-4, in the abdominal ganglion cells (iag cells), and in the lateral bipolar dendrite neurons (LBD neurons) of abdominal segments A7/A8 (Figs 2A, 2B and S5). To achieve a weaker genetic manipulation of ITP, we used the *Impl2-*GAL4 driver, which targets only a subpopulation of the ITP-producing neurons: the neurosecretory neurons in the brain (ipc-1 cells and the ipc-2a cells) and the LBD neurons in the periphery (Figs 2A, 2B and S5). To avoid potential developmental effects, we took advantage of the TARGET switch (temporal and regional gene expression targeting, [39]), by which the temperature sensitive *tubGAL80*^*ts*^ allows switching on the RNAi specifically in the adult flies. Although we did not test the RNAi efficiency in a cell-autonomous manner, the *Impl2*-based TARGET effectively decreased the global *ITP* mRNA (Fig 2C). Consistently, with targeting only a limited number of ITP-expressing neurons, the effect on the global ITP mRNA was approximately 20% weaker than the effect of the ubiquitous *daGS*-driven *ITPi* (Fig 2C). Importantly, the *ITPi* driven by *Impl2*-based TARGET was not sufficient to impair body fluids (Figs 2D and S6). Thus, this driver allowed us to disentangle the effect of *ITP* on water storage before the onset of desiccation from its role during the desiccation exposure. *ITPi* driven by the *Impl2*-based TARGET resulted in a reduced survival under desiccation (Figs 2E and S6), suggesting that *ITP* is required to cope with the desiccation stress via an additional mechanism, not only by regulating water storage prior to desiccation. An effect on desiccation survival, similar to *Impl2*-driven *ITPi*, was obtained also by *daGS*-driven *ITPi*, when the system was switched by a low RU-486 dose. This low dose (50 μM) was not sufficient to affect the body water content (Fig 2F), but was sufficient to reduce survival upon desiccation stress, although to a lower extent than the standard dose of 200 μM RU-486 (Fig 2G) used in the rest of the GeneSwitch-based experiments.

Thus, *ITP* regulates desiccation survival not only by accumulating proper levels of body water prior to the desiccation challenge, but it is also required to cope with the arid conditions.

### *ITP* signaling regulates response to osmotic stress

**Fig 3:**
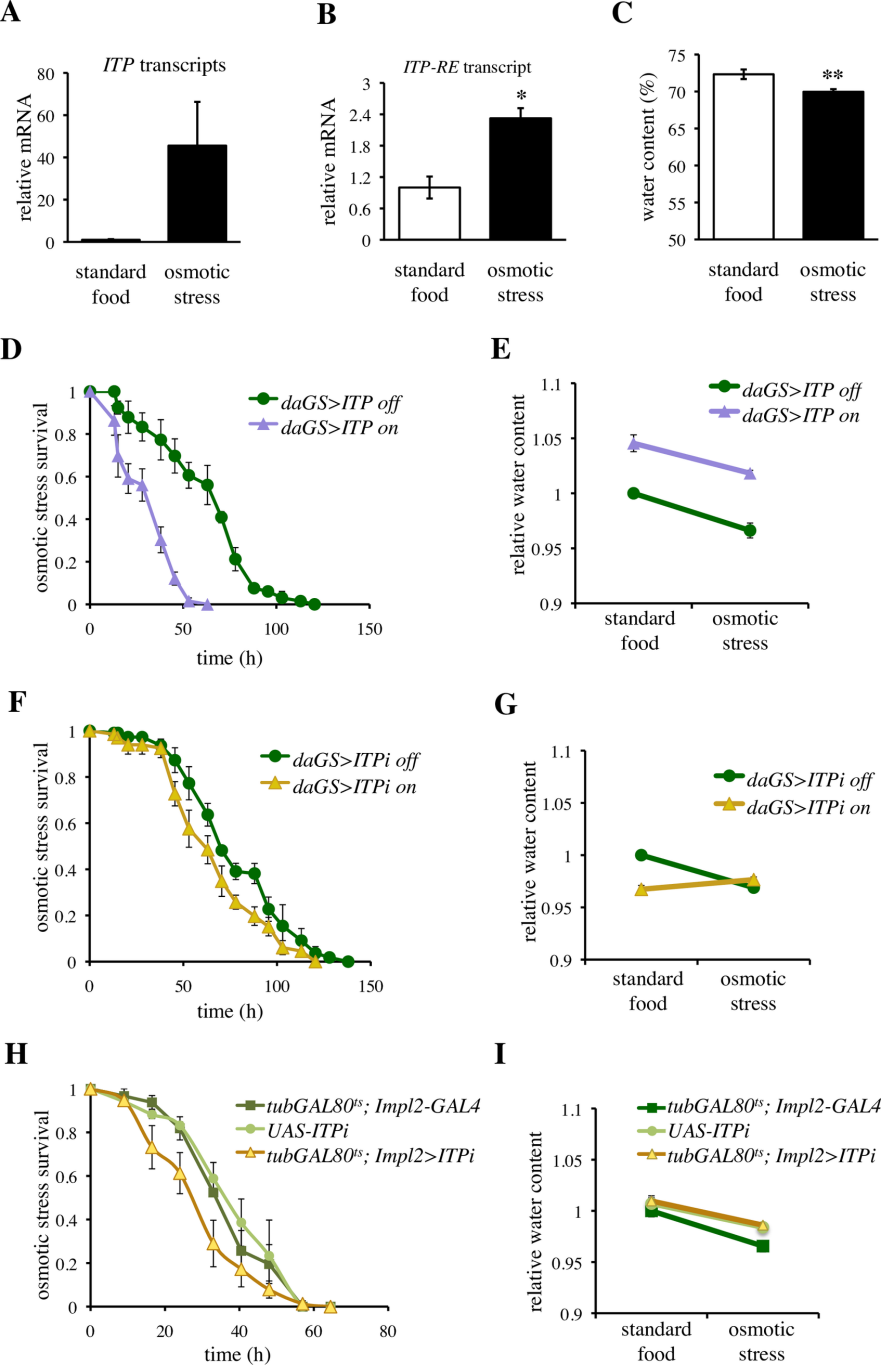
*ITP* regulates survival under osmotic stress. (A) Osmotic stress appears to increase expression of the *ITP* gene. However, when a primer pair that detects all 5 isoforms (transcripts *RC*, *RD*, *RE*, *RF* and *RG*, FlyBase FB2017_06) is used, a clear tendency is observed but the *P* value does not reach statistical significance. Two-tailed Student’s *t*–test: *P* > 0.05. (B) Osmotic stress increases abundance of the *ITP RE* transcript. A primer pair that detects solely this isoform was used. Two-tailed Student’s *t*–test: *P* < 0.05. (C) Osmotic stress decreases body fluid. Two-tailed Student’s *t*–test: *P* < 0.01. (D) Over-expression of *ITP* decreases survival during osmotic stress. Log-rank test: *P* < 0.001. Sample size: *daGS>ITP* off n = 66; *daGS>ITP* on = 66. (E) Over-expression of *ITP* increases body water, but does not affect the osmotic stress-induced loss of body water. Two-way ANOVA, *ITP* over-expression and osmotic treatment as fixed factors. Effect of *ITP*: *P* < 0.001, effect of osmotic stress: *P* < 0.001, effect of *ITP* × osmotic stress interaction: *P* > 0.05. See S1 Table for further details. (F) *daGS*-driven *ITP* RNAi (*ITPi*) decreases resistance to osmotic stress. Log-rank test: *P* < 0.05. Sample size: *daGS>ITPi* off n = 66; *daGS>ITPi* on n = 66. (G) *daGS*-driven *ITPi* decreases water content under standard conditions (two-tailed Student’s *t*–test: *P* < 0.001), and interacts with the effect of osmotic stress. Two-way ANOVA, *ITPi* and osmotic treatment as fixed factors; effect of *ITPi*: *P* < 0.01, effect of osmotic stress: *P* < 0.05, effect of the interaction: *P <* 0.001. See S2 Table for further details. (H) *ITPi* driven by the *Impl2*-based TARGET decreases osmotic stress resistance. Log-rank test: *P* < 0.01 for each comparison with controls. Sample size: *tub-GAL80*^*ts*^*; Impl2-GAL4* n = 69; *ITPi* n = 66; *tub-GAL80*^*ts*^*; Impl2>ITPi* n = 75. (I) *ITPi* driven by the *Impl2*-based TARGET does not affect water content under standard conditions (two-tailed Student’s *t*–test: *P* > 0.05 for each comparison with controls), nor interacts with the effect of osmotic stress (two-way ANOVA, *ITPi* and osmotic treatment as fixed factors; effect of the interaction: *P >* 0.05. See S3 Table for further details. Thus, the *Impl2*-based TARGET enables disentangling the requirement for *ITP* under osmotic stress from its requirement for water preservation.

Regulation of water balance is important especially under ionic stress. Therefore, we monitored *ITP* expression after feeding on a medium containing 4% NaCl, using a primer pair that covers expression of all 5 known transcripts of the gene (Fig 3A), and a primer pair specific for the *ITP-RE* transcript, the only transcript that gives rise to ITP [28]. Osmotic stress indeed increased expression of *ITP-RE* (Fig 3B). However, this treatment also reduced the amount of body water (Fig 3C) and hence we cannot differentiate whether the increase in *ITP* expression was driven by the changes in the osmolarity or the volume of body fluids. Genetic over-expression of *ITP* decreased survival during osmotic stress (Fig 3D), without affecting the osmolarity-induced changes in the body water (Fig 3E and S1 Table).

Similar to *ITP* over-expression, *ITPi* driven by the *daGS* and the *Impl2-GAL4* lines resulted in a weak, but statistically significant reduction of osmotic resistance (Fig 3F and 3H), suggesting that both up-and down-regulations of *ITP* impair osmotic tolerance. The *daGS*-driven *ITPi* reduced water levels to an extent comparable to that seen under osmotic stress (Fig 3G). Subsequent exposure to osmotic stress did not decrease the body water of the *daGS>ITPi* flies any further (Fig 3G and S2 Table). The weaker *Impl2*-driven *ITPi* neither affected water content nor its reduction by osmotic stress (Fig 3I and S3 Table), suggesting that ITP is required to cope with osmotic stress independently of the regulations of water content.

Taken together, we show that survival under osmotic challenge requires tight regulation of *ITP* expression, as both up- and down-regulation of this gene resulted in a reduced survival on a food medium with a high salt content.

### *ITP* regulates food intake

**Fig 4:**
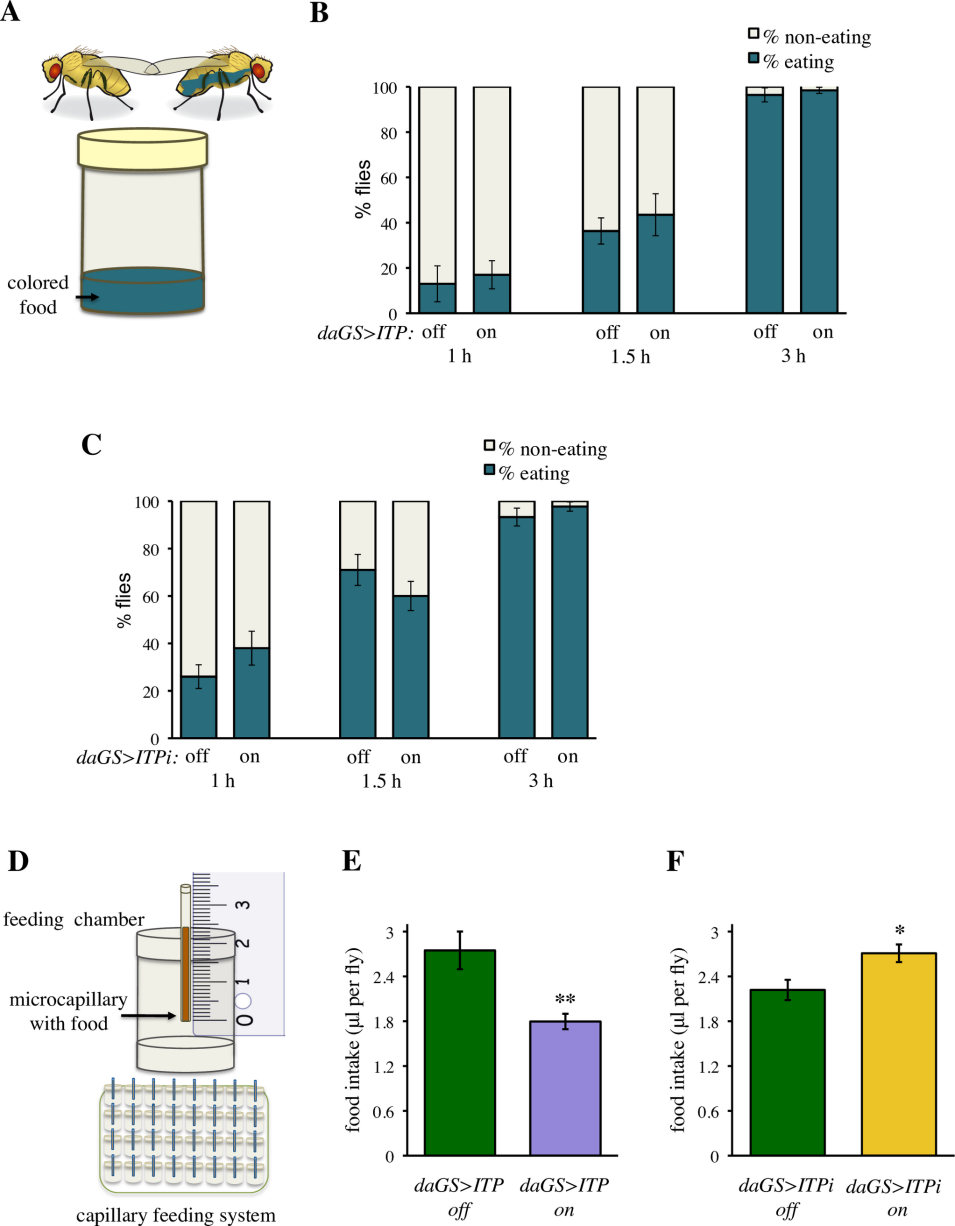
*ITP* regulates feeding. (A) Schematic drawing of the method to measure hunger as propensity to initiate feeding. (B) Over-expression of *ITP* does not affect the propensity to start feeding. Fischer’s exact test: *P* > 0.05 at all tested time points. Animals were analyzed in four replicates, Fischer’s exact test was conducted using pooled data. Sample size: *daGS>ITP* off: n = 100 (1 h), n = 96 (1.5 h), n = 78 (3 h); *daGS>ITP* on: n = 100 (1 h), n = 86 (1.5 h), n = 76 (3 h). (C) *ITPi* does not affect the propensity to start feeding. Fischer’s exact test: *P* > 0.05 at all tested time points. Animals were analyzed in four replicates, Fischer’s exact test was done using pooled data. Sample size: *daGS>ITPi* off: n = 100 (1 h), n = 100 (1.5 h), n = 87 (3 h); *daGS>ITPi* on: n = 100 (1 h), n = 100 (1.5 h), n = 85 (3 h). (D) Schematic drawing of the capillary feeding system, which measures the total volume of food eaten during a given period of time. (E) Over-expression of *ITP* decreases food intake. Two-tailed Student’s *t*–test: *P* < 0.01. (F) *ITPi* increases food intake. Two-tailed Student’s *t*–test: *P* < 0.05.

Next, we investigated the functional mechanism by which *ITP* regulates water balance. Under standard laboratory conditions, *Drosophila* obtains water from the food. Thus, we first asked whether *ITP* regulates food consumption. We tested whether *ITP* manipulations affect frequency of eating, measured as propensity to start spontaneous feeding. We transferred fed flies to fresh food supplemented with blue dye, which allows monitoring the time when animals initiate feeding (Fig 4A). Neither *ITP* over-expression nor *ITP* RNAi affected the propensity of flies to start spontaneous feeding (Fig 4B and 4C). Subsequently, we measured the total volume of food consumed (Fig 4D), using a modification of the capillary feeding (CAFE) assay [40, 41]. This assay revealed that *ITP* is an anorexigenic factor; an increase in *ITP* reduced the volume of consumed food (Fig 4E), whereas *ITP* RNAi increased the total food intake (Figs 4F and S7).

These experiments indicate that ITP is a negative regulator of food intake. Thus, increased water levels in the *daGS>ITP* and reduced levels in the *daGS>ITPi* animals suggest that ITP acts downstream of feeding to conserve body water.

### *ITP* regulates excretion

**Fig 5:**
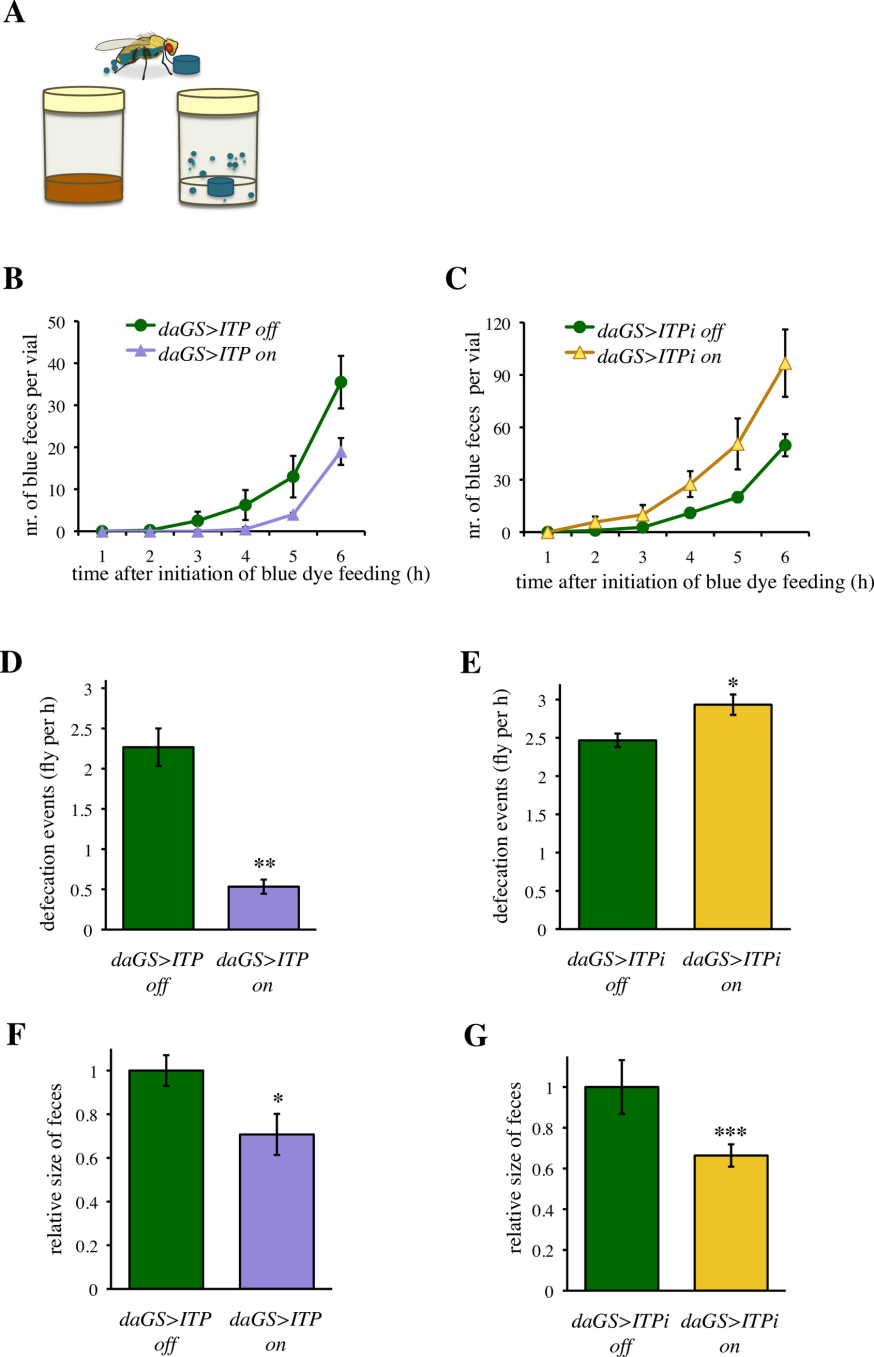
*ITP* regulates the pace of transit through the digestive tract and the number of defecation events. (A) Schematic drawing of the assay to measure defecation rate. All flies started to feed on the blue-dyed food at the same time, and the numbers of colored feces were counted every hour after the transfer on the blue dye food. (B) Over-expression of *ITP* decreases defecation rate. Two-way ANOVA, *ITP* and time as fixed factors; effect of *ITP P* < 0.01, effect of time: *P* < 0.001, effect of the interaction: *P >* 0.05. See S4 Table for further details. (C) *ITPi* increases defecation rate. Two-way ANOVA, *ITPi* and time as fixed factors; effect of *ITPi P* < 0.001, effect of time: *P* < 0.001, effect of the interaction: *P >* 0.05. See S5 Table for further details. (D) Over-expression of *ITP* decreases defecation events per fly. Two-tailed Student’s *t*–test: *P* < 0.01. (E) *ITPi* increases defecation events per fly. Two-tailed Student’s *t*–test: *P* < 0.05. (F) Over-expression of *ITP* decreases the size of feces. Two-tailed Student’s *t*–test: *P* < 0.05. (G) *ITPi* decreases the size of feces. Two-tailed Student’s *t*–test: *P* < 0.001.

The ureter of *Drosophila* feeds into the hindgut, and water that is not re-absorbed by the hindgut epithelium is excreted by the same route as the feces [9]. Thus, we investigated whether the *ITP* manipulations affect excretion. Since our previous experiments (Fig 4B and 4C) had shown that genetic manipulations of *ITP* do not affect the propensity to initiate feeding, we monitored the speed of food transit throughout the digestive tract as the time from initiation of feeding until excretion of the blue dye in the feces (Fig 5A). We transferred flies on the food with blue dye, and measured the time-dependent increase in the blue-dyed feces. The *ITP* gain-of-function reduced the speed of the food transition throughout the digestive tract (Fig 5B and S4 Table), whereas *ITP* RNAi increased it (Figs 5C and S8 and S5 Table).

Subsequently, we tested whether *ITP* regulates also the frequency of the defecation events. Thus, we continuously fed flies with the blue-dyed food for two days and observed defecation events under conditions when intake and excretion of the dye were at equilibrium. The frequency of defecation events was decreased by *ITP* over-expression (Fig 5D), and increased by *ITPi* (Fig 5E). Hence, deficiency for *ITP* leads to a phenotype reminiscent of human diarrhea. The size of individual feces was reduced by both manipulations of *ITP* (Fig 5F and 5G). Nevertheless, we were not able to detect significant differences in the color intensity of feces that might be indicative of differences in the water content (S9 Fig).

Altogether, the above experiments indicate that *ITP* regulates the rate of excretion. Deficiency in *ITP* results in a faster transit through the digestive tract and an increased number of defecation events, reminiscent of diarrhea, a common cause of dehydration in humans.

### *ITP* regulates thirst

**Fig 6:**
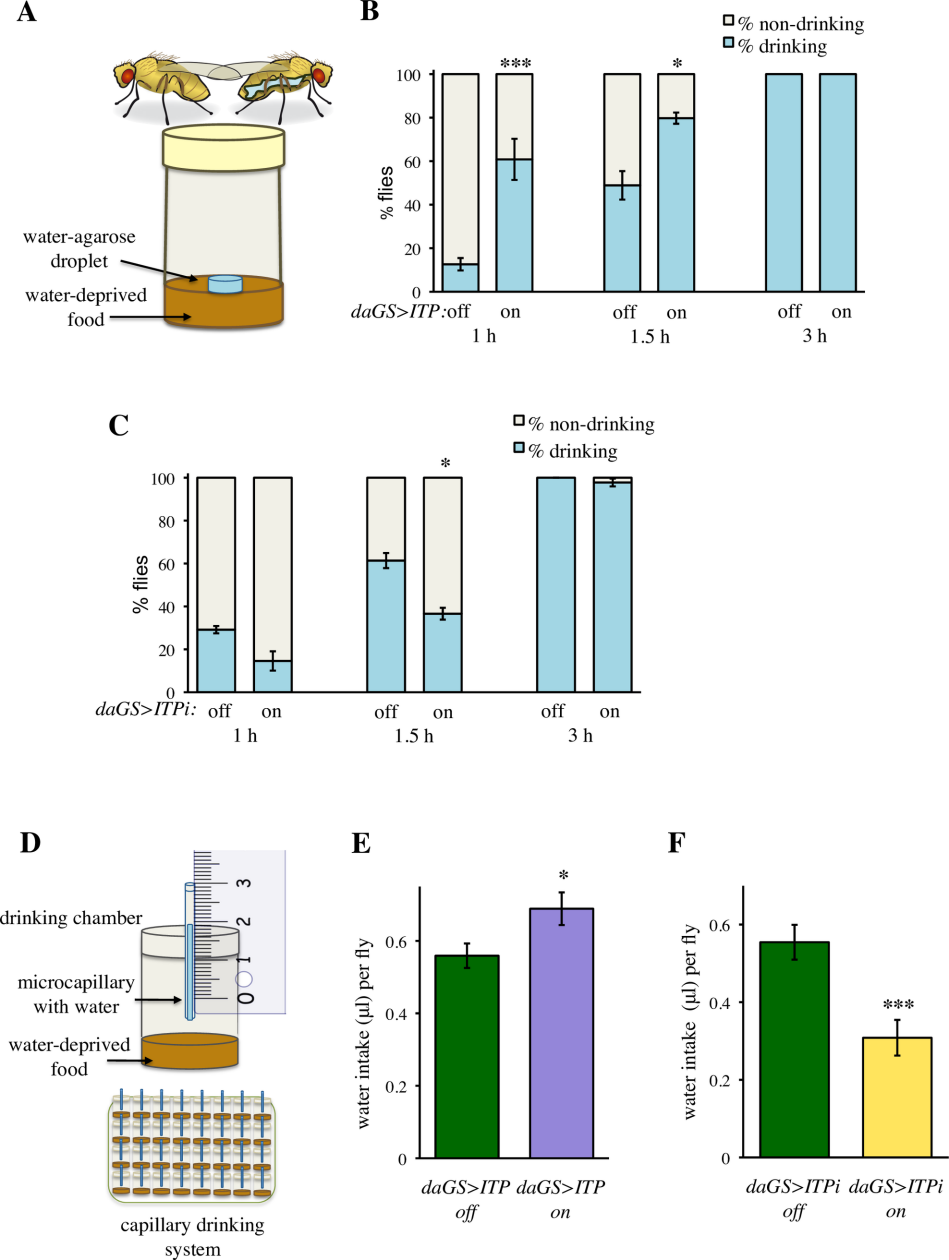
*ITP* regulates thirst and water intake. (A) Schematic drawing of a system to measure thirst as propensity to initiate water intake. (B) Over-expression of *ITP* increases thirst, i.e. reduces the time to start spontaneous drinking. Significant differences are indicated by asterisk symbols. Fischer’s exact test: * *P* < 0.05; *** *P* < 0.001. Animals were analyzed in three replicates. Fischer’s exact test was done on pooled data. Sample size: *daGS>ITP* off: n = 47 (1 h), n = 45 (1.5 h), n = 45 (3 h); *daGS>ITP* on: n = 46 (1 h), n = 44 (1.5 h), n = 43 (3 h). (C) *ITPi* reduces thirst, i.e. extends the time until flies initiate drinking. Fischer’s exact test: * *P* < 0.05. Animals were analyzed in three replicates. Fischer’s exact test was done using pooled data. Sample size: *daGS>ITPi* off: n = 48 (1 h), n = 42 (1.5 h), n = 43 (3 h); *daGS>ITPi* on: n = 48 (1 h), n = 44 (1.5 h), n = 43 (3 h). (D) Schematic drawing of the capillary drinking system, which measures the total volume of water ingested during a given period of time. Panels (E) and (F) show the mean water intake per day of feeding on a water-deprived food. (E) Over-expression of *ITP* increases the amount of ingested water. Two-tailed Student’s *t*–test: *P* < 0.05. (F) *ITPi* decreases the amount of ingested water. Two-tailed Student’s *t*–test: *P* < 0.001.

Under standard experimental conditions, flies obtain water from their food, and the classical food intake assays do not distinguish between thirst and hunger. To differentiate the role of *ITP* in water versus food intake, we modified a recent method by Lau et al. [19]. We reared flies on a medium poor in water (‘dry food’), and provided access to a separate, blue-dyed source of water (Fig 6A). Flies with increased *ITP* levels started to drink faster than controls (Fig 6B), and vice versa, *ITPi* resulted in a delayed time to the onset of water intake (Fig 6C). In order to test whether *ITP* also regulates the total volume of ingested water, we modified the CAFE assay monitors from the food intake experiment (Fig 4D); water was provided in microcapillaries in the presence of food poor in water (Fig 6D). Consistent with their increased propensity to start drinking, *ITP* over-expressing flies also drank more (Fig 6E), whereas *ITPi* flies drank less water than controls (Figs 6F and S10).

These experiments revealed that ITP is the first known hormonal regulator of thirst in *Drosophila*.

**Fig 7:**
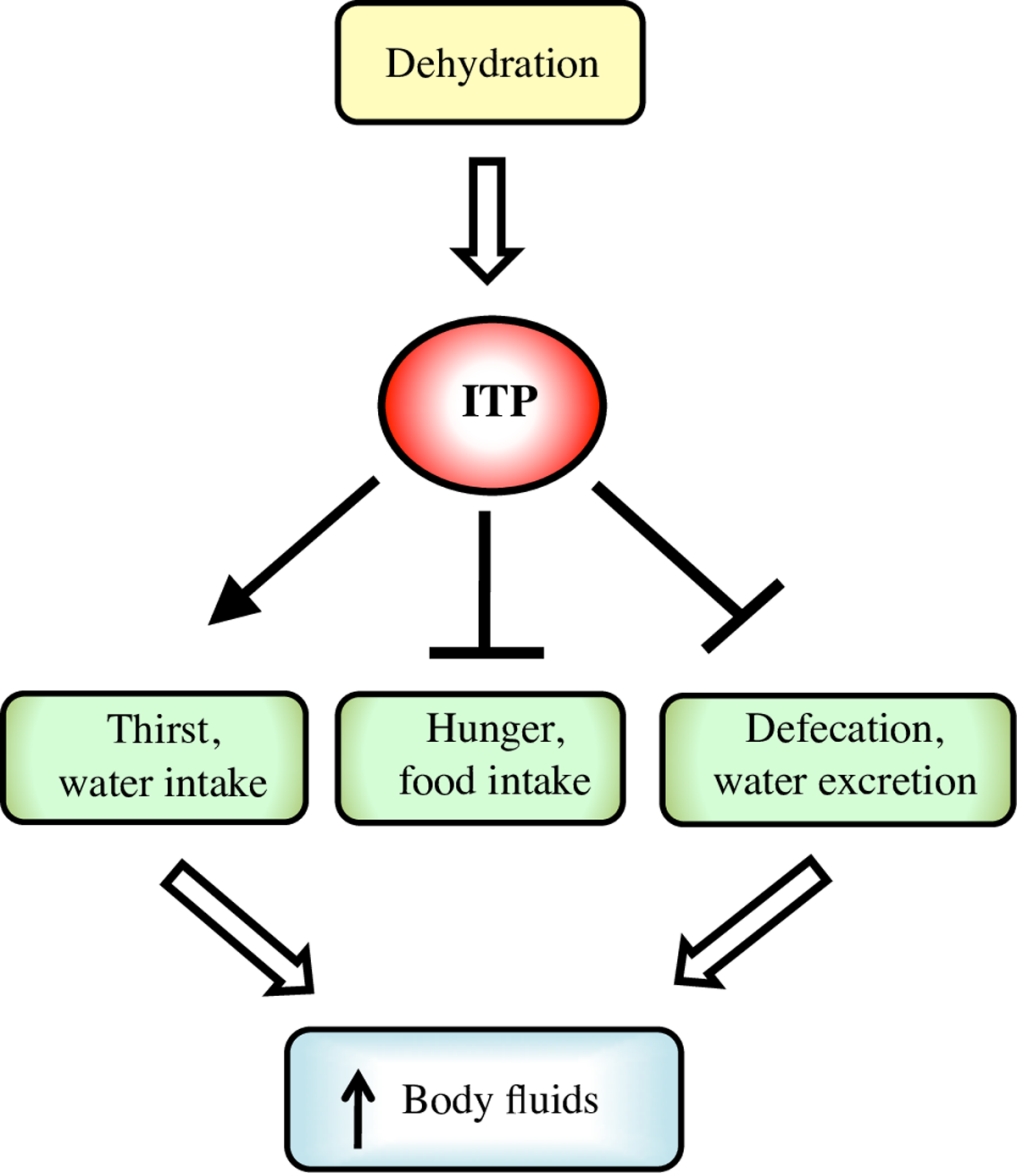
Scheme summarizing the roles of *ITP* in *Drosophila*, as revealed by this study. *ITP* is a central regulator of water balance. Conditions leading to reduced volume of body fluids result in increased expression of *ITP* gene. *ITP* subsequently promotes water intake, while inhibiting feeding and water loss by excretion, promoting thus the increase in body fluids and restoration of water balance.

In summary, in this study we identified ITP as a neuroendocrine factor central to regulation of water homeostasis. *ITP* increases in response to hypovolemia, and triggers drinking, while repressing feeding and water excretion, promoting thus conservation of water resources and protection from dehydration (Fig 7).

## Discussion

### *ITP* is a functional analog of the vasopressin and renin-angiotensin systems

With the colonization of dry land and evolution of terrestrial life, conservation, rather than elimination of water became the main challenge for the maintenance of water homeostasis [42]. Despite the differences in the organization of the endocrine systems, the main principles of fluid homeostasis are the same in vertebrates and invertebrates; these include thirst, compensation for the feeding-induced increase in osmolarity by water intake, and water re-absorption by the excretory systems [1, 9, 10, 42]. In humans, water homeostasis is regulated primarily by an osmostat located in the hypothalamus [43]. This osmostat increases water levels by triggering thirst, and reduces the water loss by inducing release of the anti-diuretic hormone vasopressin [43]. In addition to the regulation by osmolarity, thirst is also induced by the changes in the blood volume both via vasopressin [44, 45] and the renin-angiotensin system [42, 46]. Even though thirst and water retention are physiologically coupled, their regulation occurs independently [43, 47]. We show here that these regulations are simplified in *Drosophila*, where the same hormone promotes thirst, reduces appetite, and increases water storage. Thus, *ITP* acts as a functional analog of both vasopressin and renin-angiotensin. Interestingly, like the vasopressin [44, 45] and renin-angiotensin system [42, 46], also *ITP* is regulated by body water content.

Over-expression of *ITP* increases water content by 4.5%, whereas RNAi dehydrates the fly by 3.3%. The physiological consequences of such mild changes of water levels are not known in *Drosophila*, but for comparison, in human patients, loss of as little as 2% water significantly impairs cognitive abilities [48], and liquid overload and hypervolemia represent harmful conditions as well [49].

Our findings show that knockdown of *ITP* leads to increased water excretion similar to human disorders caused by defective water re-absorbance in kidney, such as diabetes insipidus [43, 50]. Conversely, *ITP* over-expression results in increased water retention reminiscent of the human syndrome of inappropriate anti-diuretic hormone secretion (SIADH) [43]. *ITP* manipulations may thus become useful tools to induce and study pathologies associated with these human disorders in *Drosophila*.

### Role of *ITP* in drinking

ITP is the first identified hormone that regulates drinking in *Drosophila*. Thus, it acts as a functional analog of the renin-angiotensin system of mammals. Similar to the renin-angiotensin system, *ITP* is most likely activated by hypovolemia. The neural circuits that control drinking and are regulated by ITP, however, remain to be investigated. Neurons that repress drinking in *Drosophila* have already been identified in the suboesophageal zone [22]. These neurons are regulated cell autonomously by an ion channel that senses osmolarity [22]. *ITP-*knockdown flies do not have the drive to drink despite their state of dehydration, whereas *ITP* over-expressing flies drink despite their excessive water content. Thus, unlike the Nanchung-expressing repressors of drinking [22], the ITP-regulated neurons are not regulated by the volume of body water, but rather by ITP itself.

### Role of *ITP* in excretion

In insects, primary urine is produced by the Malpighian tubules that are functional analogs of mammalian kidneys [9]. Water enters the lumen of these tubules by passive diffusion along the ionic gradient maintained by the vacuolar V-H^+^-ATPase [9]. The function of the Malpighian tubules is hormonally regulated by diuretic hormones [9], which in *Drosophila* include products of the genes *capa* [13, 51], *DH31* [52], *DH44* and *leucokinin* [14]. Urine then enters the hindgut, where it mixes with the gut contents. Importantly, considerable parts of the water and ions are subsequently re-absorbed in the ileum and rectum [9, 32, 53]. Here, we show that *ITP* reduces excretion of water by reducing the defection rate. Thus, it is likely that *Drosophila ITP* promotes water reabsorption in the hindgut similar to its homologs in the desert locust *Schistocerca gregaria* [31, 32] or in the European green crab *Carcinus maenas* [34]. It is noteworthy that *ITP*-expressing neurons in the abdominal ganglia innervate *Drosophila* hindgut [29], suggesting that in addition to the hormonal regulation [29], the hindgut may also be regulated by ITP in a paracrine fashion. In crabs and in the red flour beetle *Tribolium castaneum¸* CHH- or ITP*-*producing endocrine cells, respectively, have even been detected in gut epithelia [34, 54]. Thus, whether produced in the neurosecretory cells or in the endocrine cells of the gut, the actions of CHHs and ITPs on the hindgut appear to be evolutionarily conserved.

### Role of *ITP* in feeding

In mammals, an increase in osmolarity due to food intake results in postprandial thirst, and conversely, dehydration inhibits feeding when water is not available [55] and this is likely also the case in *Drosophila*. Our findings of the *ITP*-driven positive regulation of water intake, concomitant with a negative regulation of feeding likely represents another level of regulation of thirst and hunger, acting in parallel to that of the four drink-repressing neurons in the suboesophageal zone [22].

### Roles of *ITP* under desiccation and osmotic stress

Whereas many terrestrial arthropods frequently experience arid conditions, salt stress is not very common in non-blood feeding terrestrial insects. Nevertheless, desiccation and salt stress resistance have been traditional tests in the studies of *Drosophila* diuretic hormones. RNAi against diuretic hormones increases desiccation resistance, as shown for *capa* [13], *DH44* [14] and *leucokinin* [12] genes. However, it remains unclear whether these hormones contribute to the natural response to the desiccation and osmotic stress. For example, desiccation does not change expression of diuretic hormones *DH44* and *leucokinin* [14]. In contrast, *ITP* seems to be a natural component of the desiccation and osmotic stress responses, since both stressors trigger an increase in *ITP* expression. The role of ITP in thirst, hunger and excretion suggest that the ITP-regulated changes in behavior and physiology represent natural responses to cope with the reduction of body water. Consistently, knockdown of *ITP* reduces survival under desiccation and osmotic stress. However, it is unclear why over-expression of *ITP* reduces resistance to desiccation and osmotic stress. The UAS-GAL4 based manipulations may increase ITP levels far beyond the physiological range, which—although not lethal under standard feeding—might reduce survival under stressful conditions. Given the role of ITP in the ion transport across the hindgut epithelia of locusts [31, 32], it is tempting to speculate that a similar mechanism exists in *Drosophila*. In such a scenario, the non-physiological doses of ITP might considerably increase osmolarity of hemolymph. This would be toxic when feeding on a food medium with a high salt content, as well as under desiccation conditions (which further increase osmolarity).

### Possible pleiotropic actions of *ITP*, further unresolved questions and future directions

Although *ITP* has been known for a long time [56], its function has remained enigmatic in *Drosophila*. Our pioneering work on its roles in *Drosophila* physiology suggests that *ITP* codes for a master regulator of water balance, which also integrates the water homeostasis with energy metabolism. Thus, our study not only shows that this member of the CHH family has an evolutionarily conserved anti-diuretic role in *Drosophila* as it has in other arthropods [34], but also reveals novel functions of this peptide family in food and water intake. It remains to be investigated to what extent these roles are conserved in other insect species or even in crustaceans, but the strong evolutionary conservation of the gene structure [30] suggests that this might be the case. It is possible that the fly *ITP* regulates, in addition to its here-described role in water balance, other processes that are known to be CHH-regulated in crustaceans [34]. For example, the high developmental lethality of *ITP* RNAi, together with the previously described lethality of *ITP* mutants [35] imply that *Drosophila ITP* plays a critical role during development, perhaps analogous to the role of CHHs in crustacean molting [34].

Although identification of the cellular sources of ITP that are responsible for the here-described functions of this hormone was beyond the scope of this manuscript, the expression pattern of the gene already provides some tempting hints. Previous in situ-hybridizations and immunohistochemistry experiments based on a locust anti-ITP antibody showed that *Drosophila ITP* is expressed in several neuronal types [28, 29]. Here, using an antibody specific to *Drosophila* ITP, we confirmed that these cells include ipc-1 and ipc-2a neurosecretory neurons in the brain, ipc-3 and ipc-4 interneurons, three pairs of iag cells in the abdominal ganglia, and the LBD neurons in abdominal segments A7 and A8. As described previously [28, 29], although ITP is expressed in several interneurons, the most prominent cells of the brain that express ITP are the neurosecretory protocerebral ipc-1 and the ipc-2a neurons, which send axons towards neurohemal release sites in the corpora cardiaca, corpora allata, and aorta. Our experiments based on the *Impl2* driver showed that a proper response to desiccation and osmotic stress requires production of ITP in the ipc-1 neurons, ipc-2a neurons, or LBD neurons, or in their combination. The ITP production in these cells becomes nevertheless critical only under desiccation and osmotic stress. In contrast to the global manipulations, *ITPi* targeted to these neurons is not sufficient to impair water balance under standard conditions. Thus, water content is regulated either via ITP produced by cells outside of the *Impl2* expression pattern, or the ITP-producing neurons are redundant in their ability to produce sufficient ITP to maintain water homeostasis under standard conditions. Altogether, additional cell type-specific manipulations are required to differentiate whether thirst, excretion and food intake are regulated by specific neurons, or whether different ITP-producing neurosecretory cells act redundantly to produce sufficient amount of the hormone to regulate physiology of the fly.

Another key step towards understanding the ITP actions is the identification of the hitherto unknown *Drosophila* ITP receptor. This will facilitate cell- and tissue-specific manipulations to unravel the neural circuit(s) responsible for the roles of ITP in the control of thirst and hunger, and allow more detailed studies of the peripheral roles of ITP in defecation and water excretion.

## Materials & methods

### Fly lines and husbandry

Flies were reared under a 12 h light–12 h dark cycle on a standard *Drosophila* medium consisting of 6 g agar, 50 g yeast, 100 g sugar, 5.43 mL propionic acid, and 1.3 g methyl 4-hydroxybenzoate per 1 L of medium. Adult flies were collected within 24 h after eclosion, flipped on fresh media, and housed in groups of around 50 females + 50 males per vial. Flies for the TARGET experiments developed at 18°C and on the third day after eclosion were transferred to 29°C for the RNAi induction. Flies for the GeneSwitch experiments developed at 25°C on standard medium, and were kept from the third day after adult eclosion on a standard medium supplemented with RU-486 and reared further at 25°C. All GeneSwitch experiments were conducted with 0 and 200 μM RU-486, and experiments described in Fig 2F and 2G were performed also with 50 μM RU-486. After the switch induction, both TARGET and GeneSwitch flies were flipped every second day onto fresh media. If not stated otherwise, male flies were used for experiments 6–7 days after the induction of the transgene expression. Controls for the non-GeneSwitch experiments were generated by crossing the *UAS* and *GAL4* lines to the *w*^*1118*^ strain. Experiments on the desiccation and osmotic stress–induced changes in the *ITP* expression were performed on the *w*^*1118*^ strain. The list of used fly stocks is available in the S1 File.

### Viability determination

Viability was expressed as egg-to-adult survival, i.e. as the percentage of eggs that gave rise to adult flies. Three independent egg collections (each at least 120 eggs) were tested for each genotype. Eggs were counted, allowed to develop at 25°C at 12 h light/12 h dark cycle on standard medium, and eclosed flies were collected and counted.

### Water content measurements

Water content was expressed as percentage of fresh body weight. Flies were weighed using a Mettler MT5 analytical microbalance (Mettler Toledo). Fresh weight was determined, then flies were desiccated for 2 days at 65°C and weighed again. The amount of water was calculated as the difference between the fresh and the dry weight, and expressed as % of the fresh body weight. At least 5 replicates (each consisting of 5 flies) were tested per treatment / genotype.

### Desiccation resistance assay

Desiccation resistance was estimated as survival of flies in empty vials without any water source. Experiments were done in 3–4 replicates. TARGET-based experiments took place at 29°C, GeneSwitch-based experiments took place at 25°C.

### Osmotic stress assay

Osmotic stress resistance was determined as survival of flies on food medium containing 4% NaCl. Experiments were done in triplicates. TARGET-based experiments took place at 29°C, GeneSwitch-based experiments took place at 25°C. The food contained the same concentration of RU-486 (200 μM) or ethanol vehicle control as during the pre-feeding period.

### Food intake measurement by capillary feeding assay (CAFE)

The volume of ingested food was measured by a modification of the CAFE assay [40] in a feeder device constructed out of 24-well-plates, similar to the one described before [41]. Capillaries with food (Hirschmann minicaps, 5μ) were exchanged daily. Food intake of at least 15 animals per treatment was measured during 3 days, and corrected for the evaporation rate. The liquid food contained the same concentration of RU-486 (200 μM) or ethanol vehicle control as during the pre-feeding period.

### Measurement of hunger as the propensity to start feeding

Flies were transferred into a vial with a drop (approximately 0.2 mL) of food medium containing 0.5% Brilliant Blue (Sigma), and the proportion of flies that started feeding (blue dye was observable in their body after inspection under a stereomicroscope) was counted 1 h, 1.5 h and 3 h after the transfer. Flies were separated into the tested groups 1 day before the experiment to avoid potential interference of CO_2_ anesthesia with the food intake. Each time point was tested in 4 replicates, each consisting of at least 16 flies.

### Measurement of the speed of the food transit throughout the digestive tract

Flies were transferred into vials with a drop (approximately 0.2 mL) of food medium containing 0.5% Brilliant Blue (Sigma) and allowed to feed continuously. The cumulative numbers of feces that contained the blue dye were counted in the vial every hour, until 6 h after the switch to the blue-dyed medium. Feces were counted in three replicates, each vial containing 20 flies. For testing the statistical significance by two-way ANOVA, the number of new feces that were deposited within the given period was used.

### Measurement of the excretion rate at equilibrium

Excretion rate was measured as the number of defecation events (number of feces) per fly per hour. Flies were fed for 48 h on standard food (with or without RU-486) with 0.5% Brilliant Blue (Sigma). Flies were subsequently transferred into a new vial with a small drop of colored food, and the number of feces produced per fly per vial was counted. Experiments were performed in three replicates, each consisting of 20 flies.

### Analysis of the size and color intensity of the feces

Flies were fed for 48 h on standard food (with or without RU-486) with 0.5% Brilliant Blue (Sigma). Subsequently, a new transparent plastic lid was put on top of the vials, and feces collected on this lid within 2.5 h and were photographed using Leica WILD M32 stereomicroscope with Leica DFC290 camera. The area and lightness were measured using the T.U.R.D. software [57].

### Measurement of thirst / propensity to start drinking

Flies were separated into tested groups 1 day before the experiment to avoid potential effect of CO_2_ exposure on the water intake. Flies were transferred into vials containing approximately 2 mL of the water-deprived food, and after 30 min into new vials with 2 mL of the water-deprived food and a 0.2 mL of a water-rich agar droplet (0.6% agarose, 0.5% Brilliant Blue) and allowed to eat and drink. Flies that started to drink were identified based on the blue color in their abdomina after inspection under a stereomicroscope. The proportion of flies that started to drink was checked 1 h, 1.5 h and 3 h after transferring flies to the water source. Experiments were done in triplicates, and at each time point, at least 42 flies were tested. Water-deprived food medium contained 75% less water and agar than the standard medium, consisting of: 0.6 g agar, 20 g yeast, 40 g sugar, 0.54 mL propionic acid, 0.13 g methyl 4-hydroxybenzoate and 1 mL of 20 mM RU-486 or ethanol per 100 mL of medium.

### Measurement of water intake by capillary drinking assay

The capillary drinking assay was performed in a device similar to the CAFE assay feeder, with the following modifications: the bottom of each chamber contained approximately 0.8 mL of water-deprived food with 200 μM RU-486 or ethanol as a vehicle control. Water-deprived food medium contained 75% less water and agar than the standard medium, as described above. Flies were allowed to drink water from the capillaries. To make the measurements of the ingested water easier, water was colored with 0.05% Brilliant Blue (Sigma). The volume of ingested water was measured over one day, and corrected for the evaporation rate. At least 18 flies were tested for each genetic manipulation.

### Immunohistochemistry

Adult flies were dissected in ice-cold *Drosophila* Ca^2+^ free saline. After removing wings and legs, brain-thoracic/abdominal ganglia complexes were quickly excised from head and thorax. All preparations were fixed overnight in Zamboni's fixative overnight at room temperature, washed and treated as described in detail earlier [29]. The only modifications concerned the use of two different primary and secondary antibodies always at the same time of incubations. Primary antibodies were a polyclonal rabbit anti-DrmITP diluted 1:10,000 [33] and a monoclonal mouse anti-GFP (against Jelly fish GFP; Invitrogen) diluted 1:1,000. Secondary antibodies were goat anti-rabbit Alexa 546 and goat anti-mouse Alexa 488, respectively (Invitrogen), both diluted 1:1,000. Preparations were imaged with a Zeiss LSM 780 confocal microscope by use of 10× or 20× objectives. Confocal images were processed with Zeiss ZEN software, version 8.1 2012, for maximum intensity projections of z-stacks. Brightness and contrast was adjusted using Corel Photopaint X7 during plate-mounting using Corel Draw X7.

### Quantitative PCR (qPCR)

RNA was extracted using the Zymo Research QuickRNA MicroPrep kit according to the manufacturer’s instructions. cDNA was synthesized by the QuantiTect Reverse Transcription Kit (Qiagen) using 1 μg of the total RNA. Quantitative real-time PCR was performed using SensiFAST SYBR Hi-ROX Kit (Bioline) and StepOne Real-Time PCR System (Applied Biosystems). Expression levels were normalized to Actin 5C (Act5C). Information on the primers is available in the S1 File.

### Statistical analyses

Measurement variables were analyzed by two-tailed Student’s *t-*test, one-way or two-way ANOVA. Nominal variables were analyzed by two-tailed Fischer’s exact test. Survival data were analyzed by log-rank test. *P* values are indicated by asterisk symbols (* *P* < 0.05, ** *P* < 0.01, *** *P* < 0.001). Error bars represent SEM. Data on the measurement variables were analyzed using Excel or PAST [58]: http://palaeo-electronica.org/2001_1/past/issue1_01.htm. Survival data were analyzed using PAST. Data on nominal variables were analyzed by Graphpad QuickCalcs (https://www.graphpad.com/quickcalcs/).

## Supporting information

S1 Fig RU-486 compound does not affect water balance. Water content of the *w*^*1118*^ strain reared for one week on food enriched with 200 μM RU-486 does not differ from the controls reared with the carrier (ethanol). Two-tailed Student’s *t* test: *P* > 0.05.
(TIF)

S2 Fig Alternative *ITPi* line confirms the anti-diuretic effect of ITP. *ITPi* driven by an alternative RNAi strain (VDRC #43848) with a differential target region recapitulates the decrease in the proportion of body water observed with the *ITPi* line VDRC#330029 (see Fig 1F). Two-tailed Student’s *t*–test: *P* < 0.05.
(TIF)

S3 Fig ITP acts as an anti-diuretic hormone also in females. (A) Over-expression of *ITP* increases proportion of body water. Two-tailed Student’s *t*–test: *P* < 0.001. (B) *ITPi* decreases the proportion of body water. Two-tailed Student’s *t*–test: *P* < 0.01.
(TIF)

S4 Fig Genetic manipulations of *ITP* expression reduce desiccation survival also in females. (A) Over-expression of *ITP* decreases desiccation resistance. Log-rank test: *P* < 0.001. Sample size: *daGS>ITP* off n = 60; *daGS>ITP* on n = 60. (B) *ITP* RNAi decreases survival during desiccation. Log-rank test: *P* < 0.05. Sample size: *daGS>ITPi* off n = 60; *daGS>ITPi* on n = 45.
(TIF)

S5 Fig Schematic drawing of the expression pattern of *Impl2>GAL4*, *ITP*, and of their overlap. (A) Schematic drawing of the expression pattern of the *Impl2* driver (green), which covers the ipc-1 and ipc-2a cells, insulin-producing neurosecretory cells (IPCs), hugin cells (hc), cells of corpora cardiaca, some neurons in the abdominal ganglia, and the LBD neurons at the border of the dorsal abdominal segments A7/A8 next to the heart. Note that some of the *Impl2>GFP* cells in the brain and the abdominal ganglia are shown in partially reduced number for clarity and not to scale, including IPCs (around 14 cells), hugin cells (22), and adipokinetic hormone producing cells in corpora cardiaca (>8). (B) Schematic drawing of the ITP producing cells. ITP-expressing neurons that are covered by the *Impl2* driver are depicted in yellow (ipc-1 cells, ipc-2a cells and LBD neurons). Cells that express *ITP* but not *Impl2* are depicted in magenta (ipc-2, ipc-3 and ipc-4 brain neurons and the iag-cells in the abdominal ganglia).
(TIF)

S6 Fig Genetic manipulations of *ITP* by the *Impl2*- based TARGET in females recapitulates the effects of the same genetic manipulations in males. (A) *ITPi* driven by the *Impl2*-based TARGET does not affect the proportion of body water. Two-tailed Student’s *t*–test: *P* > 0.05 for both comparisons with controls. (B) *ITPi* driven by the *Impl2*-based TARGET reduces survival under desiccation. Log-rank test: *P* < 0.001 for both comparisons with controls. Sample size: *tub-GAL80*^*ts*^*; Impl2-GAL4* n = 67; *ITPi* n = 66; *tub-GAL80*^*ts*^*; Impl2-GAL4>ITPi* n = 63.
(TIF)

S7 Fig Alternative *ITPi* line confirms the anorexigenic effect of ITP. *ITPi* driven by an alternative RNAi strain (VDRC #43848) with a differential target region recapitulates the increase in food intake observed with the *ITPi* line VDRC#330029 (see Fig 4F). Two-tailed Student’s *t*–test: *P* < 0.05.
(TIF)

S8 Fig Alternative *ITPi* line confirms the role of ITP in the speed of transit through the digestive tract. *ITPi* driven by an alternative RNAi strain (VDRC #43848) with a differential target region recapitulates the increase in the defecation rate observed with the *ITPi* line VDRC#330029 (see Fig 5C). Two-way ANOVA, *ITP* and time as fixed factors; effect of *ITPi P* < 0.01, effect of time: *P* < 0.001.
(TIF)

S9 Fig Genetic manipulations of *ITP* do not lead to statistically significant changes in the color intensity / lightness of the feces. (A) Over-expression of *ITP* does not affect the lightness of feces. Two-tailed Student’s *t*–test: *P* > 0.05. (B) *ITPi* does not affect the lightness of feces. Two-tailed Student’s *t*–test: *P* > 0.05. In both (A) and (B), the measured lightness was normalized to the lightness of the controls.
(TIF)

S10 Fig Alternative *ITPi* line confirms the role of ITP in the regulation of thirst. *ITPi* driven by an alternative RNAi strain (VDRC #43848) with a differential target region recapitulates the decrease in the water intake observed with the *ITPi* line VDRC#330029 (see Fig 4F). Two-tailed Student’s *t*–test: *P* < 0.05.
(TIF)

S1 Table Additional statistical information to the legend of the Fig 3E. (TIF)

S2 Table Additional statistical information to the legend of the Fig 3G. (TIF)

S3 Table Additional statistical information to the legend of the Fig 3I. (TIF)

S4 Table Additional statistical information to legend of the Fig 5B. (TIF)

S5 Table Additional statistical information to the legend of the Fig 5C. (TIF)

S1 File List of fly stocks and primers used in the study. (PDF)

S1 Dataset (XLSX)

## References

[1] Jourjine N. Hunger and thirst interact to regulate ingestive behavior in flies and mammals. Bioessays. 2017;39(5). 10.1002/bies.201600261 28319257

[2] Baker KD, Thummel CS. Diabetic larvae and obese flies-emerging studies of metabolism in *Drosophila*. Cell Metab. 2007;6(4):257–66. 10.1016/j.cmet.2007.09.002 17908555 PMC2231808

[3] Das R, Dobens LL. Conservation of gene and tissue networks regulating insulin signalling in flies and vertebrates. Biochem Soc Trans. 2015;43(5):1057–62. 10.1042/BST20150078 26517923

[4] Owusu-Ansah E, Perrimon N. Modeling metabolic homeostasis and nutrient sensing in *Drosophila*: implications for aging and metabolic diseases. Dis Model Mech. 2014;7(3):343–50. 10.1242/dmm.012989 24609035 PMC3944494

[5] Padmanabha D, Baker KD. *Drosophila* gains traction as a repurposed tool to investigate metabolism. Trends Endocrin Met. 2014;25(10):518–27. 10.1016/j.tem.2014.03.011 24768030

[6] Beyenbach KW. The plasticity of extracellular fluid homeostasis in insects. J Exp Biol. 2016;219(Pt 17):2596–607. 10.1242/jeb.129650 27582560

[7] Gullan PSC P. J. The Insects: An Outline of Entomology, 5th edition. John Wiley & Sons, 2014. 2014.

[8] Coast GM. Neuroendocrine control of ionic homeostasis in blood-sucking insects. J Exp Biol. 2009;212(Pt 3):378–86. 10.1242/jeb.024109 19151213

[9] Gäde G. Regulation of intermediary metabolism and water balance of insects by neuropeptides. Annu Rev Entomol. 2004;49:93–113. 10.1146/annurev.ento.49.061802.123354 14651458

[10] Coast G. The endocrine control of salt balance in insects. Gen Comp Endocrinol. 2007;152(2–3):332–8. 10.1016/j.ygcen.2007.02.018 17400222

[11] Coast GM, Garside CS. Neuropeptide control of fluid balance in insects. Ann N Y Acad Sci. 2005;1040:1–8. 10.1196/annals.1327.001 15891001

[12] Zandawala M, Marley R, Davies SA, Nässel DR. Characterization of a set of abdominal neuroendocrine cells that regulate stress physiology using colocalized diuretic peptides in *Drosophila*. Cell Mol Life Sci. 2018;75(6):1099–115. 10.1007/s00018-017-2682-y 29043393 PMC5814475

[13] Terhzaz S, Teets NM, Cabrero P, Henderson L, Ritchie MG, Nachman RJ, et al Insect capa neuropeptides impact desiccation and cold tolerance. Proc Natl Acad Sci USA. 2015;112(9):2882–7. 10.1073/pnas.1501518112 25730885 PMC4352776

[14] Cannell E, Dornan AJ, Halberg KA, Terhzaz S, Dow JAT, Davies SA. The corticotropin-releasing factor-like diuretic hormone 44 (DH44) and kinin neuropeptides modulate desiccation and starvation tolerance in *Drosophila melanogaster*. Peptides. 2016;80:96–107. 10.1016/j.peptides.2016.02.004 26896569 PMC4889782

[15] Cameron P, Hiroi M, Ngai J, Scott K. The molecular basis for water taste in *Drosophila*. Nature. 2010;465(7294):91–5. 10.1038/nature09011 20364123 PMC2865571

[16] Chen ZJ, Wang QX, Wang ZR. The Amiloride-Sensitive Epithelial Na+ Channel PPK28 Is Essential for *Drosophila* Gustatory Water Reception. J Neurosci. 2010;30(18):6247–52. 10.1523/JNEUROSCI.0627-10.2010 20445050 PMC6632709

[17] Ji F, Zhu Y. A novel assay reveals hygrotactic behavior in *Drosophila*. PLoS One. 2015;10(3):e0119162 10.1371/journal.pone.0119162 25738801 PMC4349581

[18] Enjin A, Zaharieva EE, Frank DD, Mansourian S, Suh GS, Gallio M, et al Humidity Sensing in *Drosophila*. Curr Biol. 2016;26(10):1352–8. 10.1016/j.cub.2016.03.049 27161501 PMC5305172

[19] Lau MT, Lin YQ, Kisling S, Cotterell J, Wilson YA, Wang QP, et al A simple high throughput assay to evaluate water consumption in the fruit fly. Sci Rep. 2017;7(1):16786 10.1038/s41598-017-16849-6 29196744 PMC5711950

[20] Fanson BG, Yap S, Taylor PW. Geometry of compensatory feeding and water consumption in *Drosophila melanogaster*. J Exp Biol. 2012;215(Pt 5):766–73. 10.1242/jeb.066860 22323199

[21] Ja WW, Carvalho GB, Zid BM, Mak EM, Brummel T, Benzer S. Water- and nutrient-dependent effects of dietary restriction on *Drosophila* lifespan. Proc Natl Acad Sci USA. 2009;106(44):18633–7. 10.1073/pnas.0908016106 19841272 PMC2773996

[22] Jourjine N, Mullaney BC, Mann K, Scott K. Coupled Sensing of Hunger and Thirst Signals Balances Sugar and Water Consumption. Cell. 2016;166(4):855–66. 10.1016/j.cell.2016.06.046 27477513 PMC4983267

[23] Nässel DR, Winther AM. *Drosophila* neuropeptides in regulation of physiology and behavior. Prog Neurobiol. 2010;92(1):42–104. 10.1016/j.pneurobio.2010.04.010 20447440

[24] Schoofs L, De Loof A, Van Hiel MB. Neuropeptides as Regulators of Behavior in Insects. Ann Rev Entomol, Vol 62. 2017;62:35–52. 10.1146/annurev-ento-031616-035500 27813667

[25] Itskov PM, Ribeiro C. The dilemmas of the gourmet fly: the molecular and neuronal mechanisms of feeding and nutrient decision making in *Drosophila*. Front Neurosci-Switz. 2013;7 10.3389/fnins.2013.00012 23407678 PMC3569668

[26] Melcher C, Bader R, Pankratz MJ. Amino acids, taste circuits, and feeding behavior in *Drosophila*: towards understanding the psychology of feeding in flies and man. J Endocrinol. 2007;192(3):467–72. 10.1677/JOE-06-0066 17332516

[27] Pool AH, Scott K. Feeding regulation in *Drosophila*. Curr Opin Neurobiol. 2014;29:57–63. 10.1016/j.conb.2014.05.008 24937262 PMC4253568

[28] Dircksen H. Insect ion transport peptides are derived from alternatively spliced genes and differentially expressed in the central and peripheral nervous system. J Exp Biol. 2009;212(Pt 3):401–12. 10.1242/jeb.026112 19151215

[29] Dircksen H, Tesfai LK, Albus C, Nässel DR. Ion transport peptide splice forms in central and peripheral neurons throughout postembryogenesis of *Drosophila melanogaster*. J Comp Neurol. 2008;509(1):23–41. Epub 2008/04/18. 10.1002/cne.21715 18418898

[30] Webster SG, Keller R, Dircksen H. The CHH-superfamily of multifunctional peptide hormones controlling crustacean metabolism, osmoregulation, moulting, and reproduction. Gen Comp Endocrinol. 2012;175(2):217–33. 10.1016/j.ygcen.2011.11.035 22146796

[31] Audsley N, Mcintosh C, Phillips JE. Actions of Ion-transport peptide from locust corpus cardiacum on several hindgut transport processes. J Exp Biol. 1992;173:275–88 10.1242/jeb.173.1.261 1487715

[32] Phillips JE, Wiens C, Audsley N, Jeffs L, Bilgen T, Meredith J. Nature and control of chloride transport in insect absorptive epithelia. J Exp Zool. 1996;275(4):292–9. 10.1002/(SICI)1097-010X(19960701)275:4<292::AID-JEZ7>3.0.CO;2-K 8759926

[33] Hermann-Luibl C, Yoshii T, Senthilan PR, Dircksen H, Helfrich-Förster C. The ion transport peptide is a new functional clock neuropeptide in the fruit fly *Drosophila melanogaster*. J Neurosci. 2014;34(29):9522–36. 10.1523/JNEUROSCI.0111-14.2014 25031396 PMC6608324

[34] Chung JS, Dircksen H, Webster SG. A remarkable, precisely timed release of hyperglycemic hormone from endocrine cells in the gut is associated with ecdysis in the crab *Carcinus maenas*. Proc Natl Acad Sci USA. 1999;96(23):13103–7. 10.1073/pnas.96.23.13103 10557280 PMC23907

[35] Park Y, Kim, H., Li, D., Adams, M. A Novel function of ion transport peptide in Drosophila ecdysis. Program and Abstracts 45th Annual Drosophila Research Conference, Washington, DC 2004;Abstract 774C (Flybase ID: FBrf0174159).

[36] Osterwalder T, Yoon KS, White BH, Keshishian H. A conditional tissue-specific transgene expression system using inducible GAL4. Proc Natl Acad Sci USA. 2001;98(22):12596–601. 10.1073/pnas.221303298 11675495 PMC60099

[37] Roman G, Endo K, Zong L, Davis RL. P[Switch], a system for spatial and temporal control of gene expression in *Drosophila melanogaster*. Proc Natl Acad Sci U S A. 2001;98(22):12602–7. 10.1073/pnas.221303998 11675496 PMC60100

[38] Tricoire H, Battisti V, Trannoy S, Lasbleiz C, Pret AM, Monnier V. The steroid hormone receptor EcR finely modulates *Drosophila* lifespan during adulthood in a sex-specific manner. Mech Ageing Dev. 2009;130(8):547–52. 10.1016/j.mad.2009.05.004 19486910

[39] McGuire SE, Le PT, Osborn AJ, Matsumoto K, Davis RL. Spatiotemporal rescue of memory dysfunction in *Drosophila*. Science. 2003;302(5651):1765–8. 10.1126/science.1089035 14657498

[40] Ja WW, Carvalho GB, Mak EM, de la Rosa NN, Fang AY, Liong JC, et al Prandiology of *Drosophila* and the CAFE assay. Proc Natl Acad Sci USA. 2007;104(20):8253–6. 10.1073/pnas.0702726104 17494737 PMC1899109

[41] Gáliková M, Klepsatel P, Xu YJ, Kühnlein RP. The obesity-related adipokinetic hormone controls feeding and expression of neuropeptide regulators of *Drosophila* metabolism. Eur J Lipid Sci Tech. 2017;119(3). 10.1002/ejlt.201600138

[42] Danziger J, Zeidel ML. Osmotic homeostasis. Clin J Am Soc Nephrol. 2015;10(5):852–62. 10.2215/CJN.10741013 25078421 PMC4422250

[43] Verbalis JG. Disorders of water metabolism: diabetes insipidus and the syndrome of inappropriate antidiuretic hormone secretion. Handb Clin Neurol. 2014;124:37–52. 10.1016/B978-0-444-59602-4.00003-4 25248578

[44] Arima H, Kondo K, Kakiya S, Nagasaki H, Yokoi H, Yambe Y, et al Rapid and sensitive vasopressin heteronuclear RNA responses to changes in plasma osmolality. J Neuroendocrinol. 1999;11(5):337–41. 10320560 10.1046/j.1365-2826.1999.00308.x

[45] Hayashi M, Arima H, Goto M, Banno R, Watanabe M, Sato I, et al Vasopressin gene transcription increases in response to decreases in plasma volume, but not to increases in plasma osmolality, in chronically dehydrated rats. Am J Physiol Endocrinol Metab. 2006;290(2):E213–7. 10.1152/ajpendo.00158.2005 16144818

[46] Fitzsimons JT. Angiotensin, thirst, and sodium appetite. Physiol Rev. 1998;78(3):583–686. 10.1152/physrev.1998.78.3.583 9674690

[47] Baylis PH, Thompson CJ. Osmoregulation of vasopressin secretion and thirst in health and disease. Clin Endocrinol. 1988;29(5):549–76. 10.1111/j.1365-2265.1988.tb03704.x PubMed PMID: WOS:A1988Q717500010. 3075528

[48] Grandjean AC, Grandjean NR. Dehydration and cognitive performance. J Am Coll Nutr. 2007;26(5 Suppl):549S–54S 17921464 10.1080/07315724.2007.10719657

[49] McGuire MD, Heung M. Fluid as a Drug: Balancing Resuscitation and Fluid Overload in the Intensive Care Setting. Adv Chronic Kidney D. 2016;23(3):152–9. 10.1053/j.ackd.2016.02.006 27113691

[50] Lu HA. Diabetes Insipidus. Adv Exp Med Biol. 2017;969:213–25. 10.1007/978-94-024-1057-0_14 28258576

[51] Davies SA, Cabrero P, Povsic M, Johnston NR, Terhzaz S, Dow JA. Signaling by *Drosophila* capa neuropeptides. Gen Comp Endocrinol. 2013;188:60–6. 10.1016/j.ygcen.2013.03.012 23557645

[52] Coast GM, Webster SG, Schegg KM, Tobe SS, Schooley DA. The *Drosophila melanogaster* homologue of an insect calcitonin-like diuretic peptide stimulates V-ATPase activity in fruit fly Malpighian tubules. J Exp Biol. 2001;204(10):1795–804 11316500 10.1242/jeb.204.10.1795

[53] Phillips JE. Excretion in insects: function of gut and rectum in concentrating and diluting the urine. Fed Proc. 1977;36(11):2480–6 20337

[54] Begum K, Li B, Beeman RW, Park Y. Functions of ion transport peptide and ion transport peptide-like in the red flour beetle *Tribolium castaneum*. Insect Biochem Mol Biol. 2009;39(10):717–25. 10.1016/j.ibmb.2009.08.005 19715761

[55] Zimmerman CA, Leib DE, Knight ZA. Neural circuits underlying thirst and fluid homeostasis. Nat Rev Neurosci. 2017;18(8):459–69. 10.1038/nrn.2017.71 28638120 PMC5955721

[56] Hewes RS, Taghert PH. Neuropeptides and neuropeptide receptors in the *Drosophila* melanogaster genome. Genome research. 2001;11(6):1126–42 10.1101/gr.169901 11381038 PMC311076

[57] Wayland MT, Defaye A, Rocha J, Jayaram SA, Royet J, Miguel-Aliaga I, et al Spotting the differences: probing host/microbiota interactions with a dedicated software tool for the analysis of faecal outputs in *Drosophila*. J Insect Physiol. 2014;69:126–35. 10.1016/j.jinsphys.2014.05.023 24907675 PMC4194350

[58] Hammer Ø, Harper DAT, Ryan PD. PAST: Paleontological statistics software package for education and data analysis. Palaeontologia Electronica. 2001;4(1). http://palaeo-electronica.org/2001_1/past/issue1_01.htm

[59] Dethier VG. The Hungry Fly A Physiological Study of the Behavior Associated with Feeding. Harvard University Press, 1974

