# Supplementary figures and images for: The thirsty fly: Ion transport peptide (ITP) is a novel endocrine regulator of water homeostasis in Drosophila
Source: PLoS Genet. 2018 Aug 23;14(8):e1007618. doi: 10.1371/journal.pgen.1007618 (PMC6124785; doi:10.1371/journal.pgen.1007618)

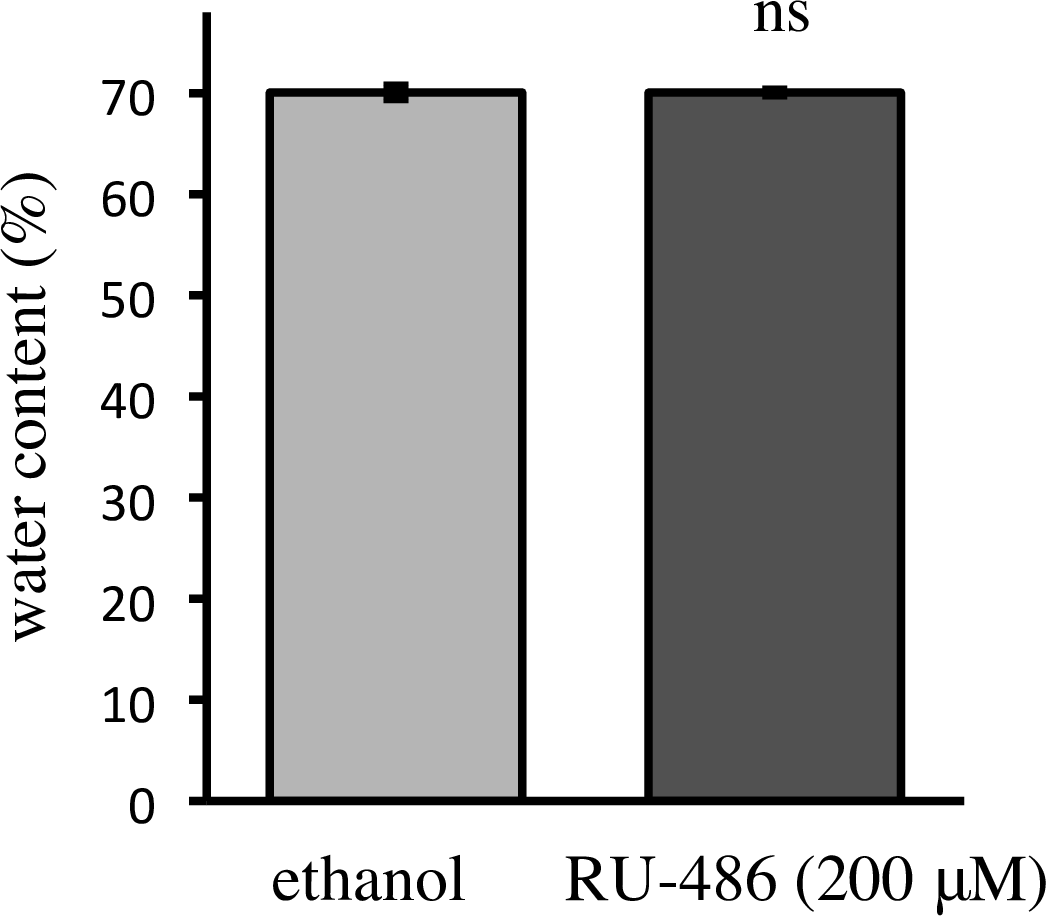

Supplement: S1 Fig — Water content of the w1118 strain reared for one week on food enriched with 200 μM RU-486 does not differ from the controls reared with the carrier (ethanol). Two-tailed Student’s t test: P > 0.05. (TIF) [file pgen.1007618.s001.tif]

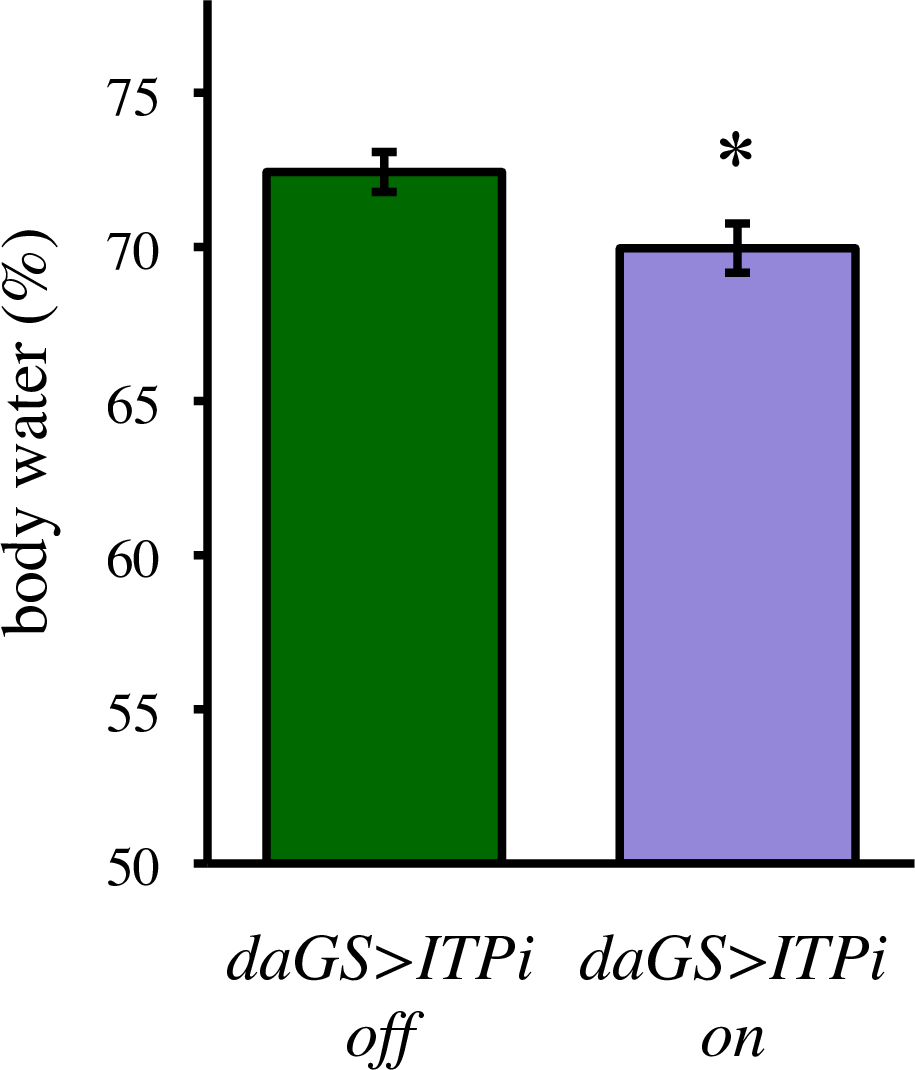

Supplement: S2 Fig — ITPi driven by an alternative RNAi strain (VDRC #43848) with a differential target region recapitulates the decrease in the proportion of body water observed with the ITPi line VDRC#330029 (see Fig 1F). Two-tailed Student’s t–test: P < 0.05. (TIF) [file pgen.1007618.s002.tif]

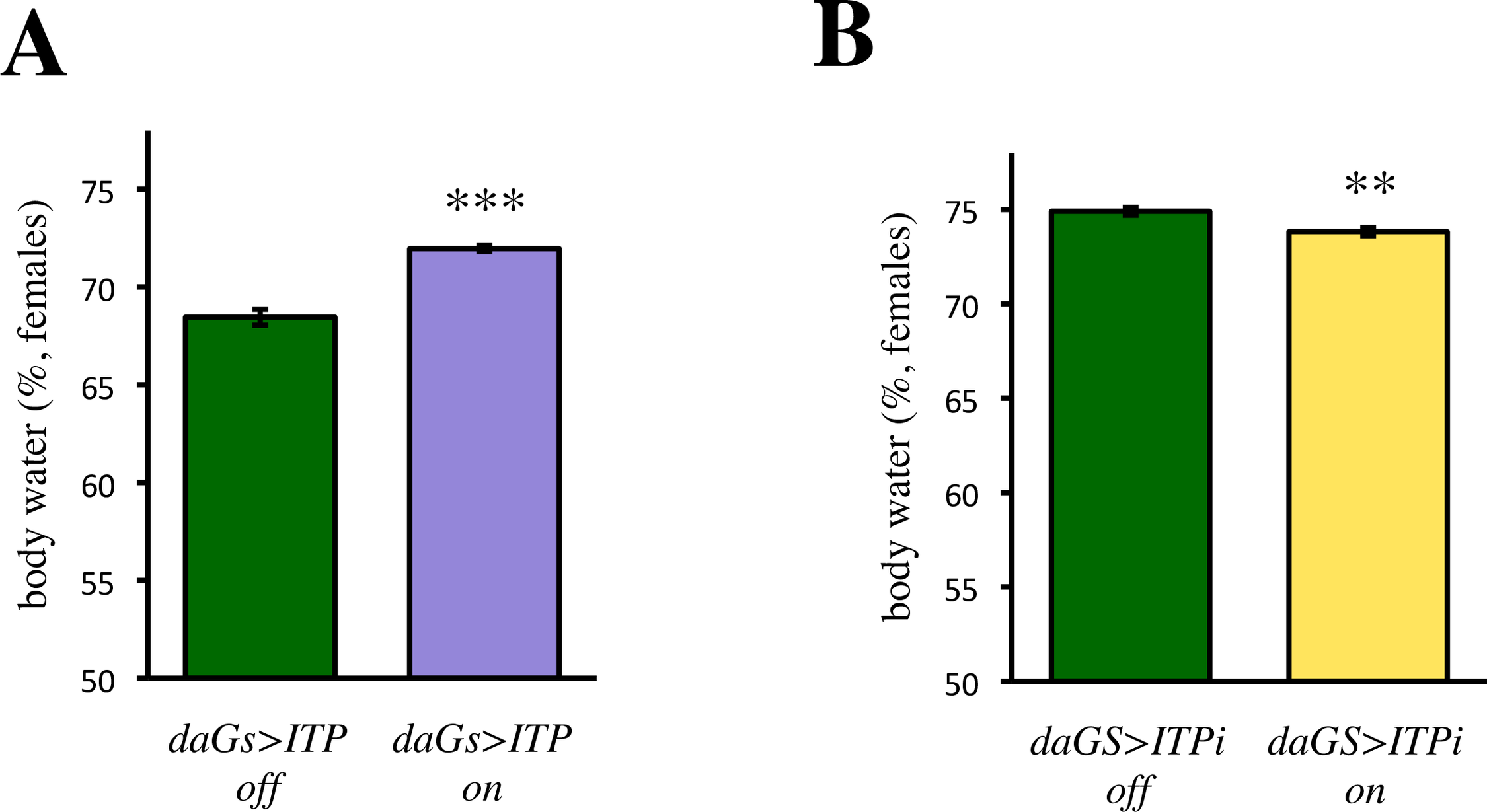

Supplement: S3 Fig — (A) Over-expression of ITP increases proportion of body water. Two-tailed Student’s t–test: P < 0.001. (B) ITPi decreases the proportion of body water. Two-tailed Student’s t–test: P < 0.01. (TIF) [file pgen.1007618.s003.tif]

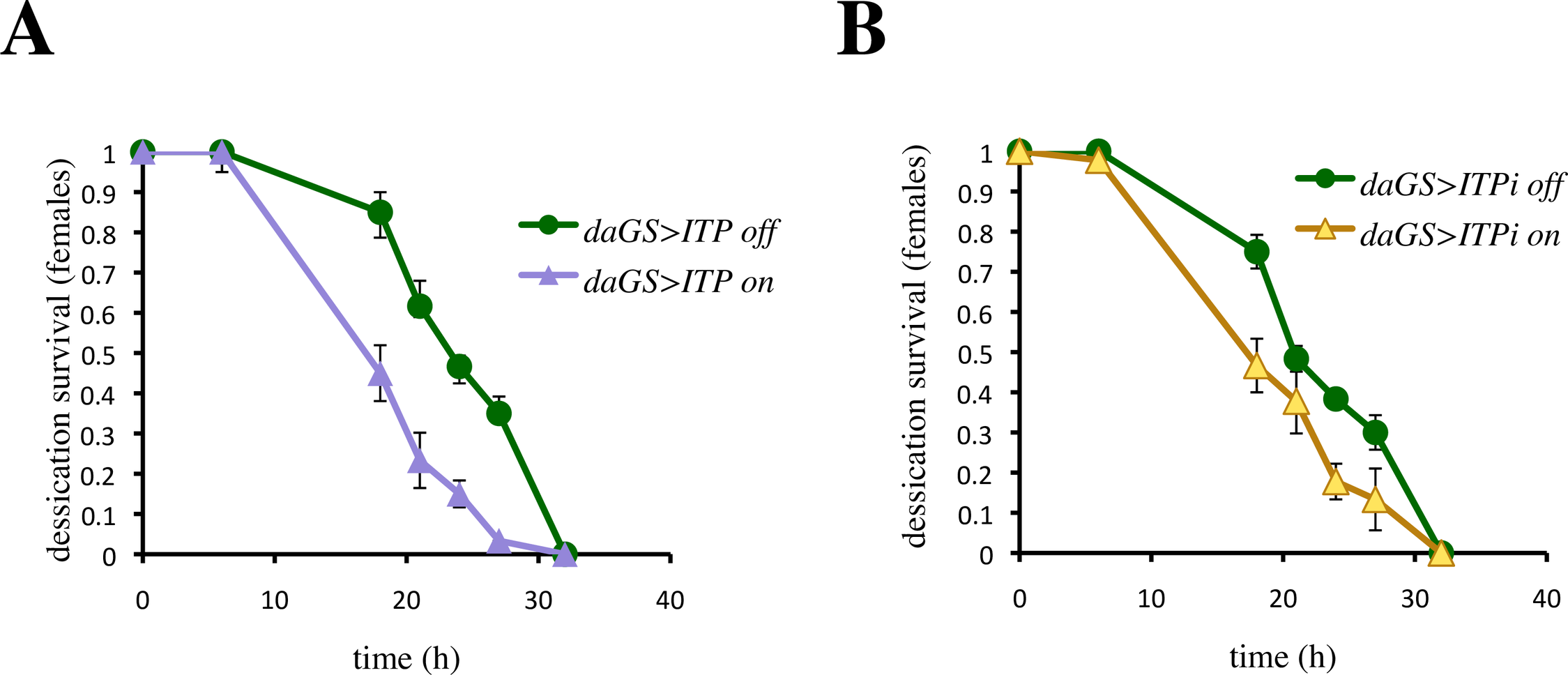

Supplement: S4 Fig — (A) Over-expression of ITP decreases desiccation resistance. Log-rank test: P < 0.001. Sample size: daGS>ITP off n = 60; daGS>ITP on n = 60. (B) ITP RNAi decreases survival during desiccation. Log-rank test: P < 0.05. Sample size: daGS>ITPi off n = 60; daGS>ITPi on n = 45. (TIF) [file pgen.1007618.s004.tif]

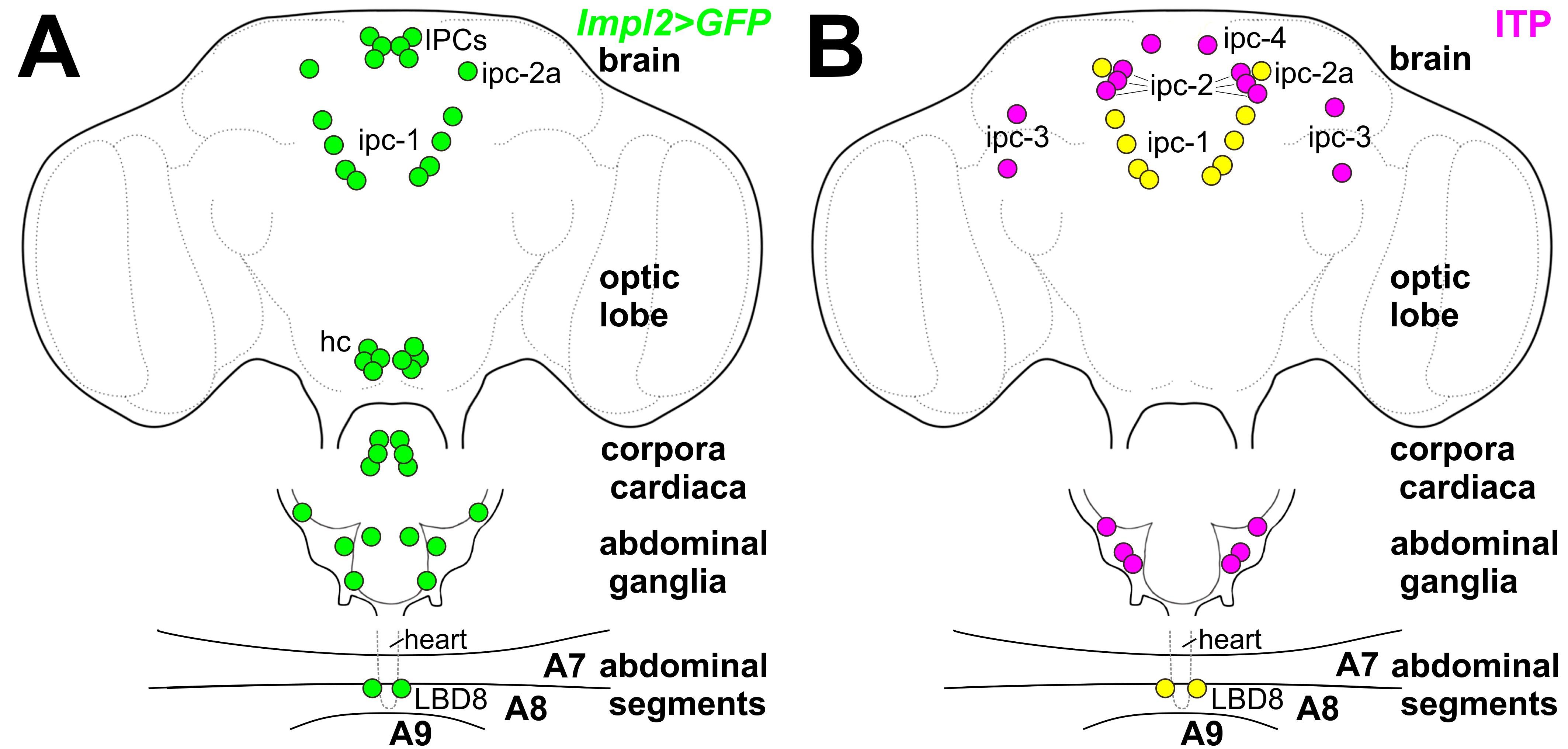

Supplement: S5 Fig — (A) Schematic drawing of the expression pattern of the Impl2 driver (green), which covers the ipc-1 and ipc-2a cells, insulin-producing neurosecretory cells (IPCs), hugin cells (hc), cells of corpora cardiaca, some neurons in the abdominal ganglia, and the LBD neurons at the border of the dorsal abdominal segments A7/A8 next to the heart. Note that some of the Impl2>GFP cells in the brain and the abdominal ganglia are shown in partially reduced number for clarity and not to scale, including IPCs (around 14 cells), hugin cells (22), and adipokinetic hormone producing cells in corpora cardiaca (>8). (B) Schematic drawing of the ITP producing cells. ITP-expressing neurons that are covered by the Impl2 driver are depicted in yellow (ipc-1 cells, ipc-2a cells and LBD neurons). Cells that express ITP but not Impl2 are depicted in magenta (ipc-2, ipc-3 and ipc-4 brain neurons and the iag-cells in the abdominal ganglia). (TIF) [file pgen.1007618.s005.tif]

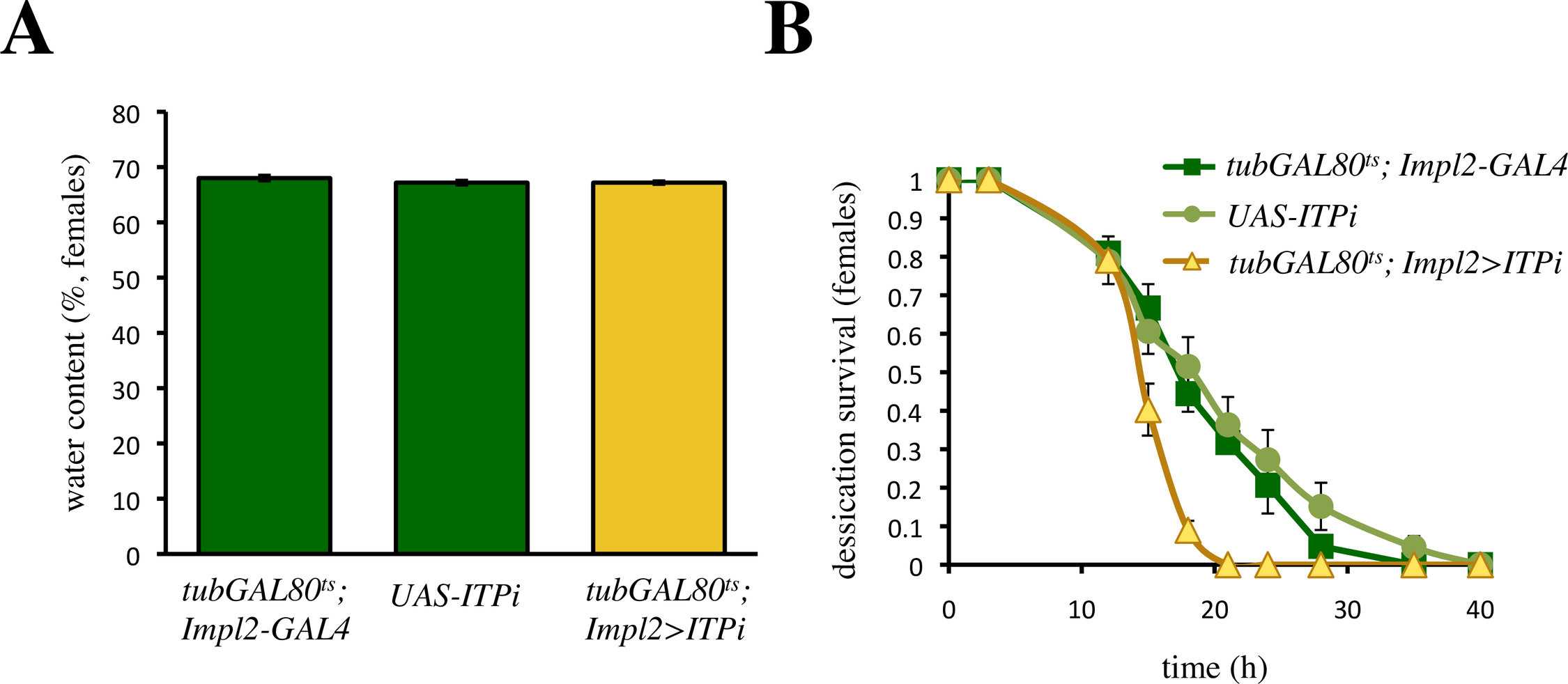

Supplement: S6 Fig — (A) ITPi driven by the Impl2-based TARGET does not affect the proportion of body water. Two-tailed Student’s t–test: P > 0.05 for both comparisons with controls. (B) ITPi driven by the Impl2-based TARGET reduces survival under desiccation. Log-rank test: P < 0.001 for both comparisons with controls. Sample size: tub-GAL80ts; Impl2-GAL4 n = 67; ITPi n = 66; tub-GAL80ts; Impl2-GAL4>ITPi n = 63. (TIF) [file pgen.1007618.s006.tif]

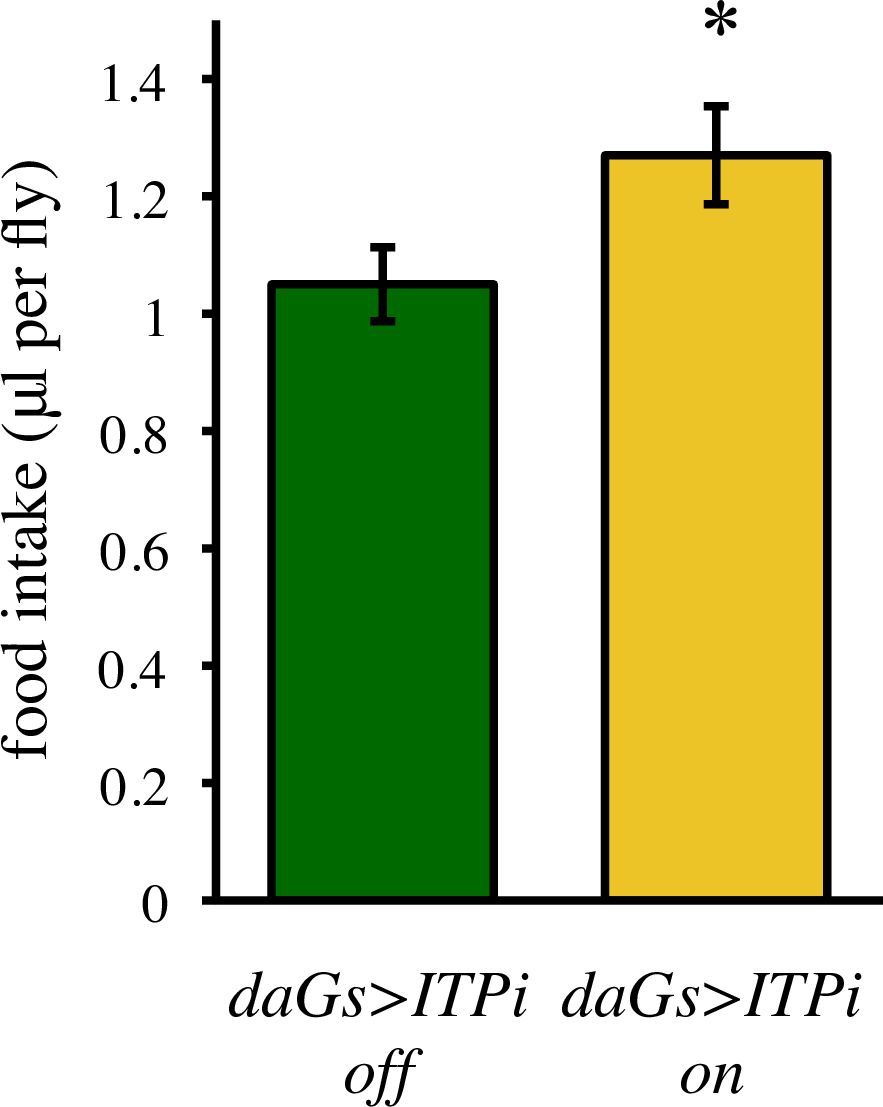

Supplement: S7 Fig — ITPi driven by an alternative RNAi strain (VDRC #43848) with a differential target region recapitulates the increase in food intake observed with the ITPi line VDRC#330029 (see Fig 4F). Two-tailed Student’s t–test: P < 0.05. (TIF) [file pgen.1007618.s007.tif]

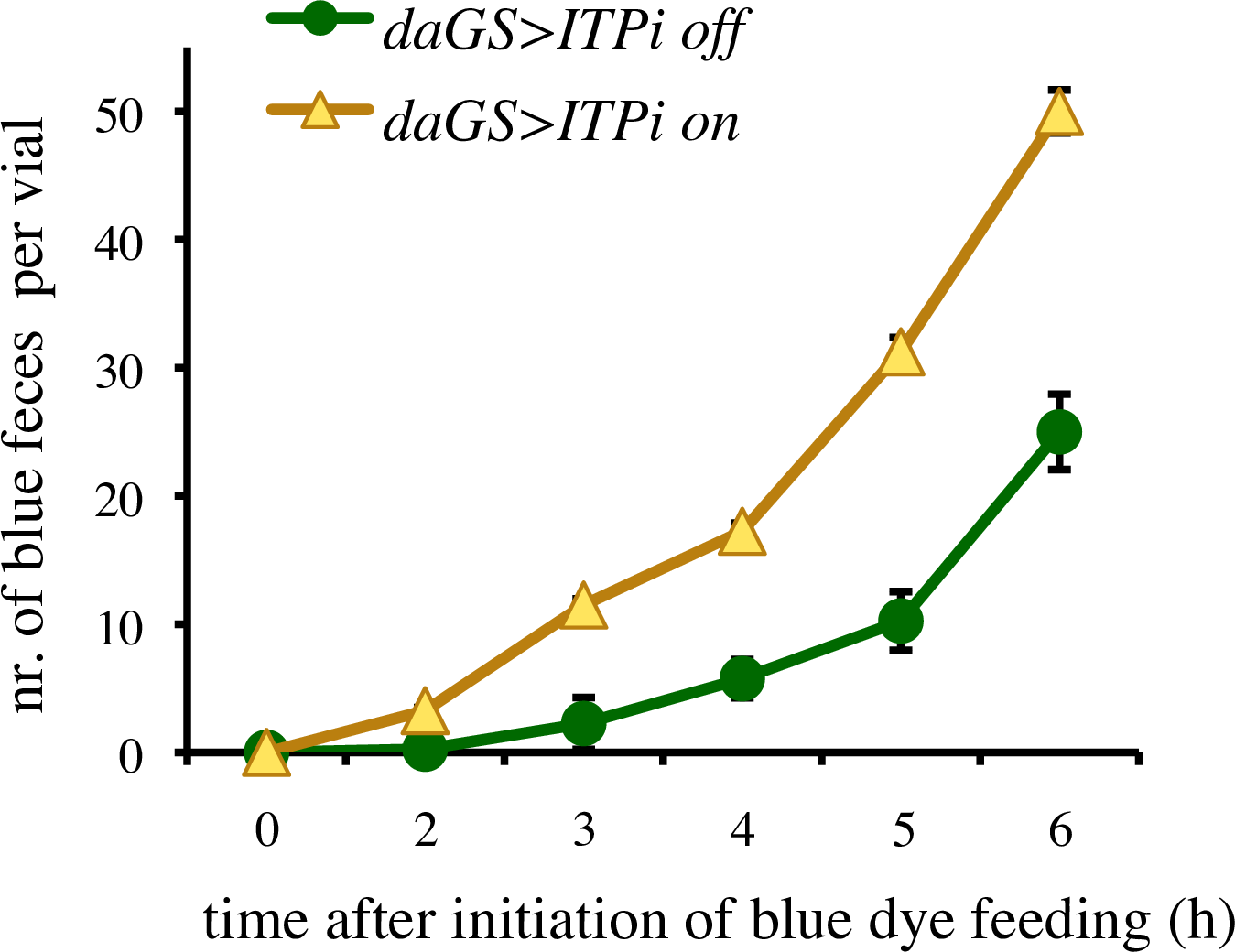

Supplement: S8 Fig — ITPi driven by an alternative RNAi strain (VDRC #43848) with a differential target region recapitulates the increase in the defecation rate observed with the ITPi line VDRC#330029 (see Fig 5C). Two-way ANOVA, ITP and time as fixed factors; effect of ITPi P < 0.01, effect of time: P < 0.001. (TIF) [file pgen.1007618.s008.tif]

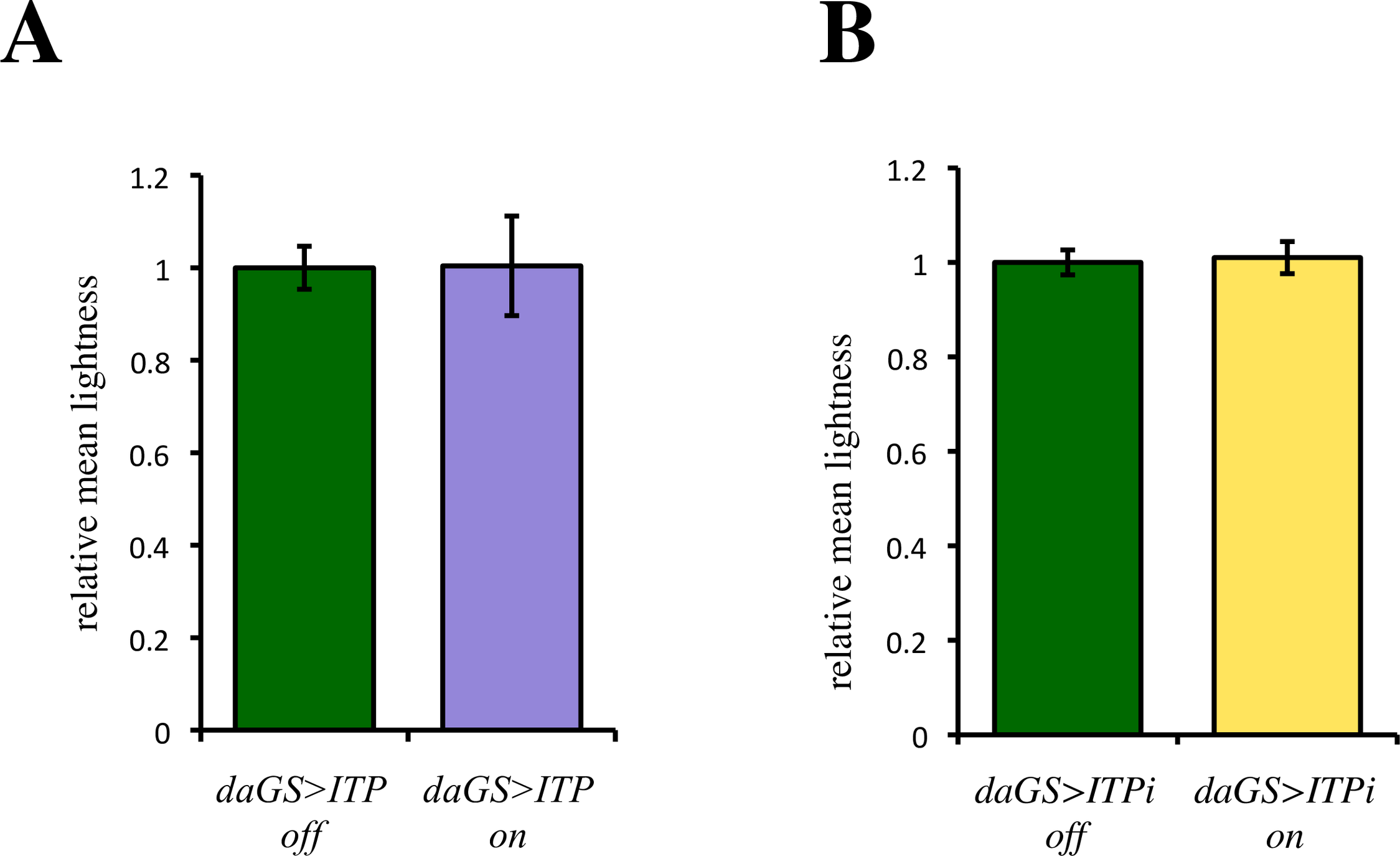

Supplement: S9 Fig — (A) Over-expression of ITP does not affect the lightness of feces. Two-tailed Student’s t–test: P > 0.05. (B) ITPi does not affect the lightness of feces. Two-tailed Student’s t–test: P > 0.05. In both (A) and (B), the measured lightness was normalized to the lightness of the controls. (TIF) [file pgen.1007618.s009.tif]

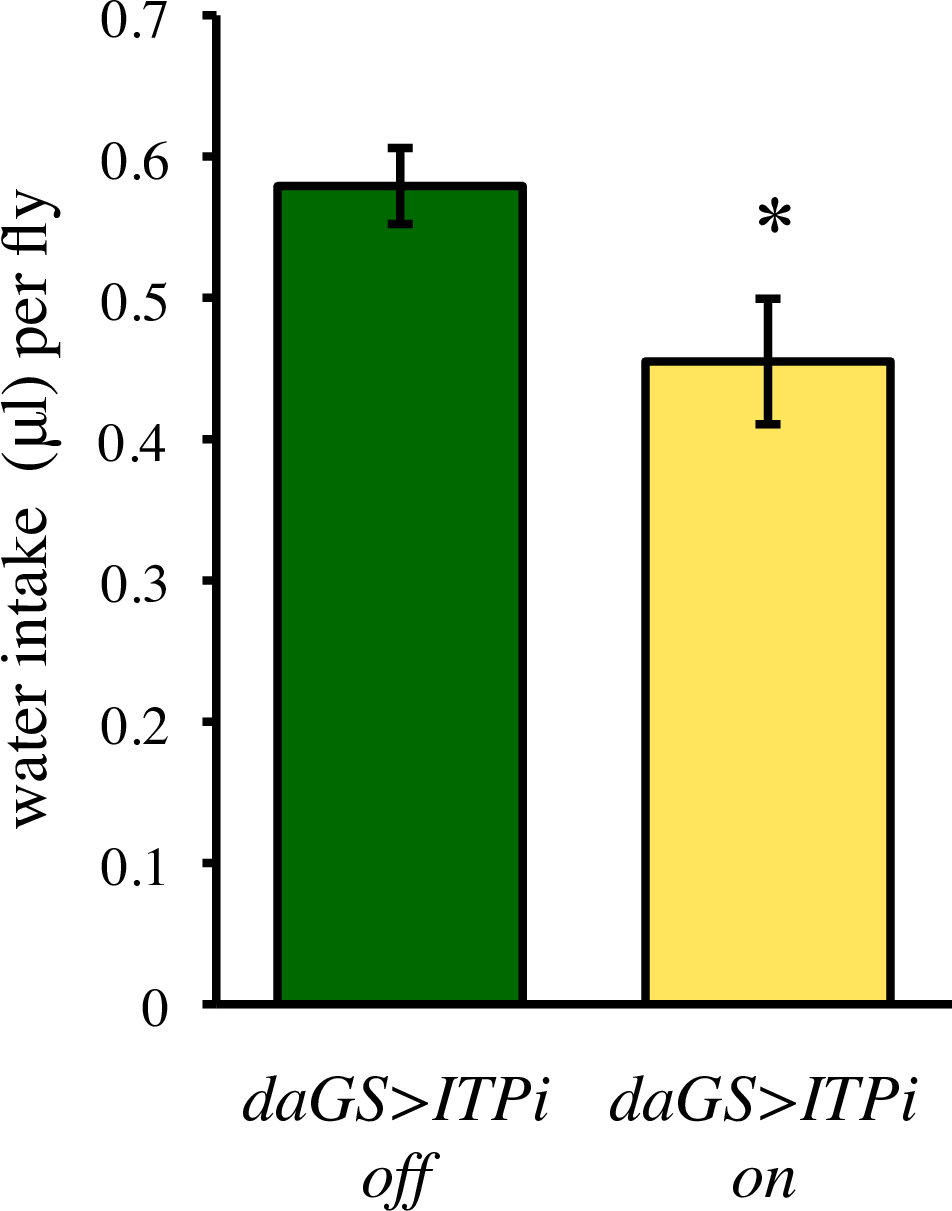

Supplement: S10 Fig — ITPi driven by an alternative RNAi strain (VDRC #43848) with a differential target region recapitulates the decrease in the water intake observed with the ITPi line VDRC#330029 (see Fig 4F). Two-tailed Student’s t–test: P < 0.05. (TIF) [file pgen.1007618.s010.tif]

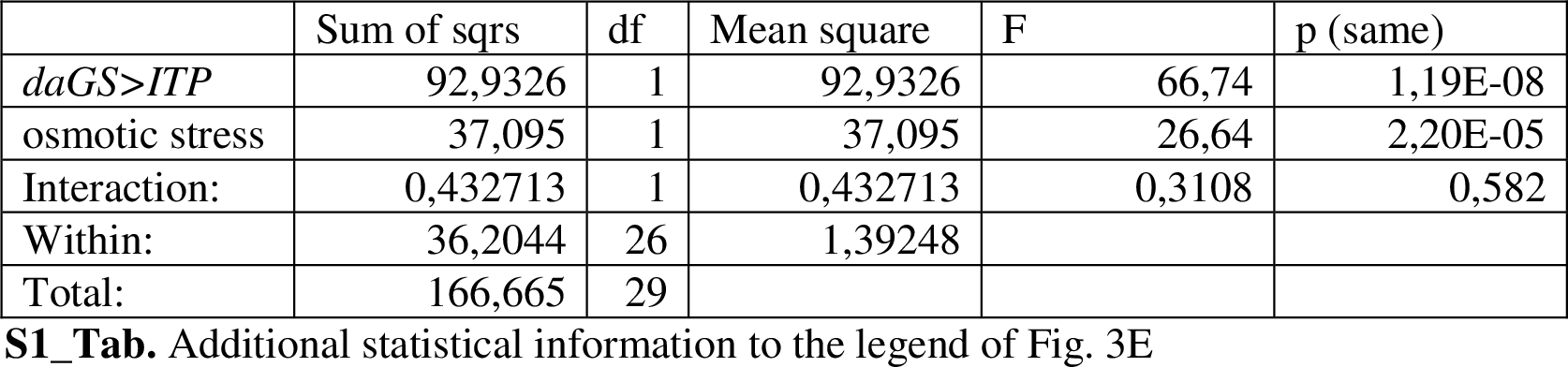

Supplement: S1 Table — (TIF) [file pgen.1007618.s011.tif]

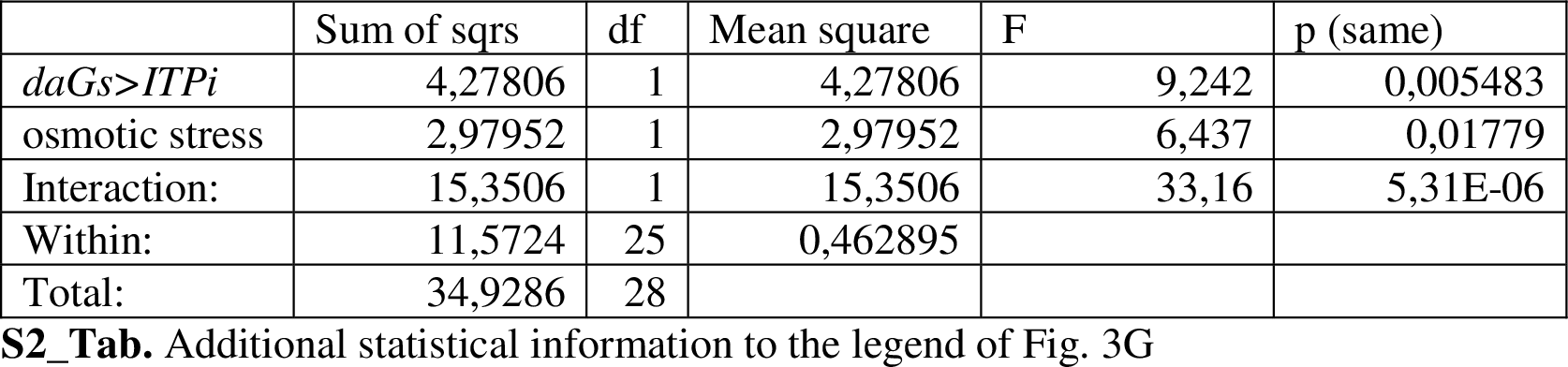

Supplement: S2 Table — (TIF) [file pgen.1007618.s012.tif]

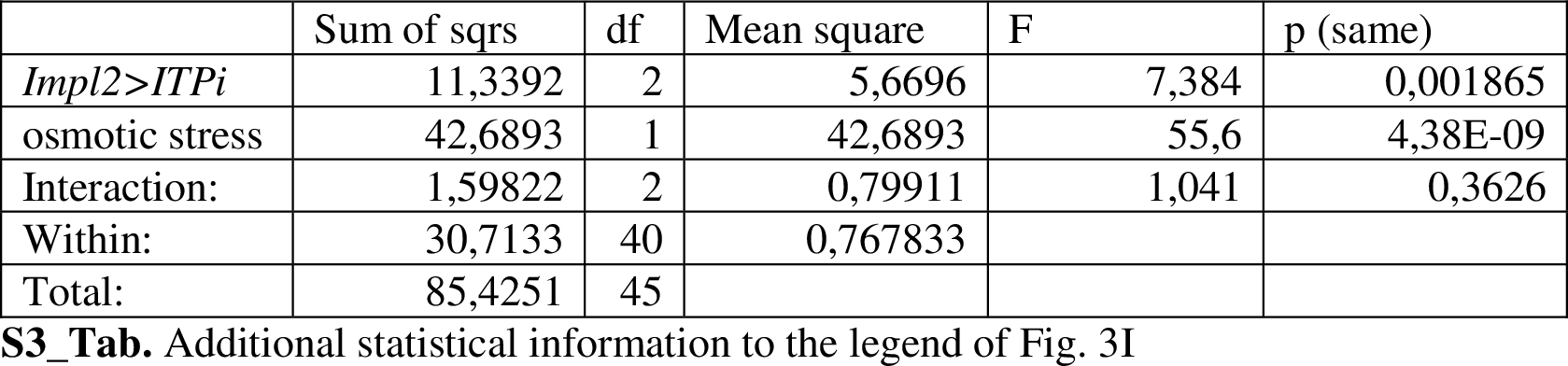

Supplement: S3 Table — (TIF) [file pgen.1007618.s013.tif]

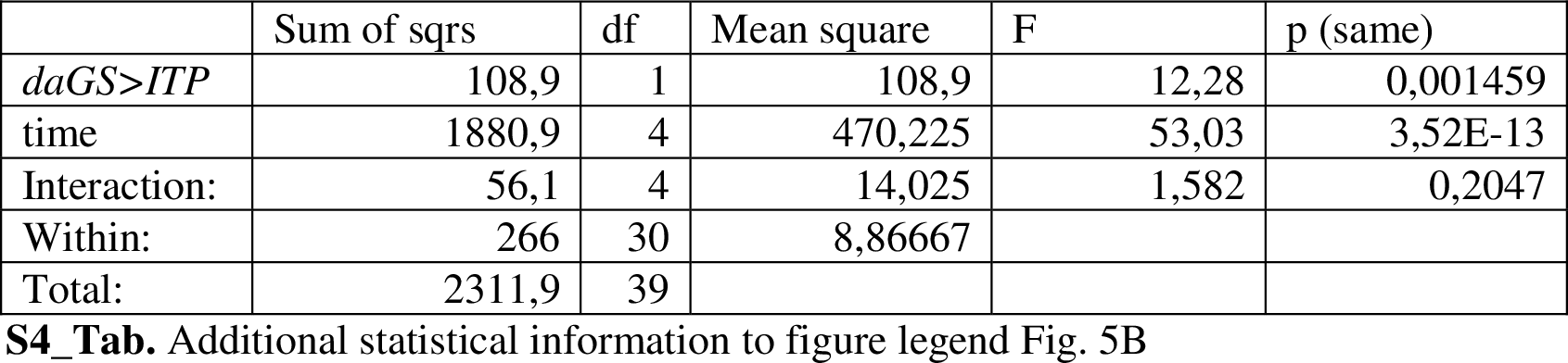

Supplement: S4 Table — (TIF) [file pgen.1007618.s014.tif]

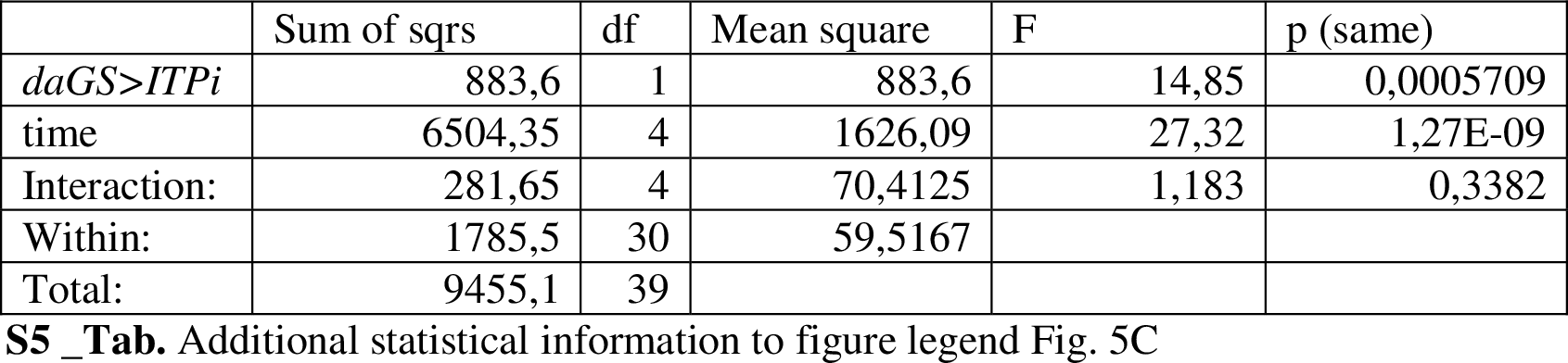

Supplement: S5 Table — (TIF) [file pgen.1007618.s015.tif]
