# Supplementary material for: The thirsty fly: Ion transport peptide (ITP) is a novel endocrine regulator of water homeostasis in Drosophila
Source: PLoS Genet. 2018 Aug 23;14(8):e1007618. doi: 10.1371/journal.pgen.1007618 (PMC6124785; doi:10.1371/journal.pgen.1007618)
Supplement: S1 File — (PDF) [file pgen.1007618.s016.pdf]

List of fly lines used in the study

Genotype	External stock number / reference
w^{1118}	VDRC#60000
w^{1118}; *daughterless-GeneSwitch*	[1]
w^{1118}; *UAS-ITP / (CyO)*	[2]
w^{1118}; *UAS-ITPi / (Tm3 Ser*^{1}*)*	VDRC#43848
w^{1118}; *UAS-ITPi*	VDRC#330029
yw; + ; Impl2-RA-GAL4 / TM6b, Tb Hu	[3]
w^{1118}; *tubGAL80*^{ts}*/(CyO); Impl2-RA-GAL4 /Tm6 Tb Hu*	This study (*Impl2*-based TARGET line)
w^{1118}; *daughterless-GeneSwitch, UAS-ITP /(CyO)*	
w^{1118}; *daughterless-GeneSwitch, UAS-ITPi /(CyO)*	
w^{1118}; *daughterless-GeneSwitch / (CyO); UAS-ITPi /(Tm3 Ser*^{1}*)*	
w; 20xUAS-IVS-mCD8::GFP*	BDRC#32194
w; tubGAL80*^{ts}*; TM2/TM6B, Tb*^{1}	BDRC#7108

List of oligonucleotides used in the study

Gene	Sequence	Reference
ITP (all transcripts)	F:CACAATCTGTCGAAGCGCAG<br>R:GGTGTATACTCGTCTCGCGG	FlyPrimerBank [4]
ITP (*ITP-RE*)	F: ATTATGCAAGCAAGAATGCTTCGG<br>R:AAATCCCAGAGAATCGCACTTTAC	This study
Act5C	F: GTGCACCGCAAGTGCTTCTAA<br>R: TGCTGCACTCCAAACTTCCAC	[5]

References

1. Tricoire H, Battisti V, Trannoy S, Lasbleiz C, Pret AM, Monnier V. The steroid hormone receptor EcR finely modulates *Drosophila* lifespan during adulthood in a sex-specific manner. Mech Ageing Dev. 2009;130(8):547-52. doi: 10.1016/j.mad.2009.05.004

2. Hermann-Luibl C, Yoshii T, Senthilan PR, Dircksen H, Helfrich-Forster C. The Ion Transport Peptide Is a New Functional Clock Neuropeptide in the Fruit Fly *Drosophila melanogaster*. J Neurosci. 2014;34(29):9522-36. doi: 10.1523/Jneurosci.0111-14.2014

3. Bader R, Sarraf-Zadeh L, Peters M, Moderau N, Stocker H, Kohler K, et al. The IGFBP7 homolog Imp-L2 promotes insulin signaling in distinct neurons of the *Drosophila* brain. J Cell Sci. 2013;126(Pt 12):2571-6. doi: 10.1242/jcs.120261

4. Hu Y, Sopko R, Foos M, Kelley C, Flockhart I, Ammeux N, et al. FlyPrimerBank: an online database for *Drosophila melanogaster* gene expression analysis and knockdown evaluation of RNAi reagents. G3 (Bethesda). 2013;3(9):1607-16. doi: 10.1534/g3.113.007021

5. Galikova M, Klepsatel P, Munch J, Kuhnlein RP. Spastic paraplegia-linked phospholipase PAPLA1 is necessary for development, reproduction, and energy metabolism in *Drosophila*. Sci Rep. 2017;7:46516. doi: 10.1038/srep46516
